# Combined treatment with Metformin and 2-deoxy glucose induces detachment of viable MDA-MB-231 breast cancer cells *in vitro*

**DOI:** 10.1038/s41598-017-01801-5

**Published:** 2017-05-11

**Authors:** Maruša Bizjak, Petra Malavašič, Klemen Dolinar, Jelka Pohar, Sergej Pirkmajer, Mojca Pavlin

**Affiliations:** 1 Group for nano and biotechnological applications, Faculty of Electrical Engineering, University of Ljubljana, Ljubljana, Slovenia; 2 Institute of Pathophysiology, Faculty of Medicine, University of Ljubljana, Ljubljana, Slovenia; 3 Department of Synthetic Biology and Immunology, National institute of Chemistry, Ljubljana, Slovenia; 4 Centre of Excellence EN-FIST, Ljubljana, Slovenia; 5 Institute of Biophysics, Faculty of Medicine, University of Ljubljana, Ljubljana, Slovenia

## Abstract

Triple naegative breast cancer has an increased rate of distant metastasis and consequently poor prognosis. To metastasize, breast cancer cells must detach from the main tumour mass and resist anoikis, a programmed cell death induced by lack of cell-extracellular matrix communication. Although cancer cells must detach to metastasize *in vivo*, the viability of floating cancer cells *in vitro* is rarely investigated. Here we show that co-treatment of anoikis-resistant MDA-MB-231 cells with metformin and 2-deoxy-D-glucose (2-DG) increased the percentage of floating cells, of which about 95% were viable. Floating cells resumed their proliferation once they were reseeded in the pharmacological compound-free medium. Similar effects on detachment were observed on anoikis-prone MCF-7 cells. Co-treatment of MDA-MB-231 cells with metformin and 2-DG induced a strong activation of AMP-activated protein kinase (AMPK), which was reduced by AMPK inhibitor compound C that prevented detachment of MDA-MB-231 cells. However, direct AMPK activators A-769662 and AICAR did not have any major effect on the percentage of floating MDA-MB-231 cells, indicating that AMPK activation is necessary but not sufficient for triggering detachment of cancer cells. Our results demonstrate that separate analysis of floating and attached cancer cells might be important for evaluation of anti-cancer agents.

## Introduction

Triple negative breast cancer, characterised by the absence of estrogen receptor, progesterone receptor and human epidermal growth factor receptor 2 (HER-2), has a poor prognosis mostly due to increased rate of distant metastases^1, 2^. During the process of metastasation, cancer cells in primary tumour locally invade the tumour-associated stroma, detach from the invasion front of the tumour, and enter the lymphatic and/or blood vessels. Circulating cancer cells ultimately migrate through the capillary wall in distant tissues, re-attach to the extracellular matrix, and proliferate in a new microenvironment^3^. Once cancer cells detach from the main tumour mass, they must resist anoikis, a programmed cell death induced by extracellular matrix detachment^4^. MDA-MB-231 cells, the most commonly used *in vitro* model of triple negative breast cancer^5^, are highly metastatic and tumorigenic^5^. They form colonies in an anchorage-independent condition^6^, and are resistant to anoikis^7^. Albeit breast cancer cells must detach from extracellular matrix in order to metastasise *in vivo* ^8–11^, floating MDA-MB-231 cells *in vitro* are commonly thought to be dead. Only a few studies investigated the viability of floating MDA-MB-231 cells *in vitro* ^12–14^.

Metabolic adaptations enable survival of cancer cells in an anchorage-independent condition^6, 15–18^. By stimulating glucose uptake, oncogenes restore redox and energy balance and prevent anoikis in breast cancer cells^18^. Another way to prevent anoikis in cancer cells is by antioxidant treatment or through the upregulation of NADPH producing pathways^15, 18^. Interestingly, various cancer cells grown in an anchorage-independent condition utilize reductive carboxylation of glutamine-derived carbon to mitigate mitochondrial ROS production^16^. Redox and energy stress induced upon detachment of cancer cells also activates AMP-activated protein kinase (AMPK)^15, 17^, which regulates energy homeostasis by suppressing anabolic and enhancing catabolic processes^19^. While supraphysiological AMPK activation suppresses growth of fast proliferating cancer cells^20, 21^, it enables survival of non-proliferating cancer cells in unfavourable conditions by stimulating fatty acid oxidation through inhibition of acetyl-CoA carboxylases (ACC)^15^. Moreover, several studies show that AMPK activation regulates cell adhesion^22–24^. However, the role of AMPK activation in the process of breast cancer cell detachment is currently unknown.

Metformin and 2-deoxy glucose (2-DG) are two of the most commonly used AMPK activators. Metformin, an oral antidiabetic drug, is being evaluated in multiple clinical trials as an adjuvant drug to chemotherapy. Retrospective pharmaco-epidemiological studies on diabetic patients suggest that metformin prolongs survival for some types of breast cancers^25^, while it has no substantial effect on triple negative breast cancer^25, 26^. Metformin improves systemic glucose homeostasis, which lowers the level of insulin, a well-known mitogen for insulin-sensitive breast cancer cells^27^. However, metformin can also target cancer cells directly^28, 29^ by binding to complex I in mitochondrial respiratory chain, thus suppressing oxidative phosphorylation^30, 31^. Furthermore, metformin induces reductive carboxylation of glutamine-derived carbon in cancer cells^32–34^, which mitigates mitochondrial nutrient metabolism and increases their reliance on glycolysis^33^. 2-DG was also assessed in several clinical studies as an anticancer agent^35–37^ and is commonly used *in vitro* to mimic glucose starvation. Inhibition of glycolysis is likely its main mechanism of action, although recent studies show that 2-DG may have also non-specific effects^15, 38–42^. Therefore, metformin and 2-DG generate energy crisis, which increases concentrations of AMP and activates AMPK^43^. AMPK activation is augmented, when cancer cells are treated with both compounds simultaneously^44–46^. However, although combined treatment with both compounds synergistically suppresses proliferation of cancer cells, it does not necessarily kill them^45, 46^.

In the present study, we have found that combined treatment with metformin and low concentrations of 2-DG induces detachment of adherent MDA-MB-231 cells from the bottom of standard cell culture plates *in vitro*. Surprisingly, the vast majority of the floating MDA-MB-231 cells were viable and proliferated upon re-seeding in a fresh cell culture medium without pharmacological treatments. Anoikis-prone MCF-7 cells were also detached by co-treatment with 2-DG and metformin; however, only about 29% of floating MCF-7 were dead. Furthermore, we show that cell detachment is partially regulated by AMPK activation, although AMPK activation per se does not increase the percentage of floating cells. Taken together, our results show that combined treatment of MDA-MB-231 cells with metformin and 2-DG results in substantial number of viable floating cells, which is in contrast to commonly accepted belief that the floating cells *in vitro* are dead.

## Results

### Combined treatment with metformin and 2-DG induces detachment of MDA-MB-231 cells

**Figure 1:**
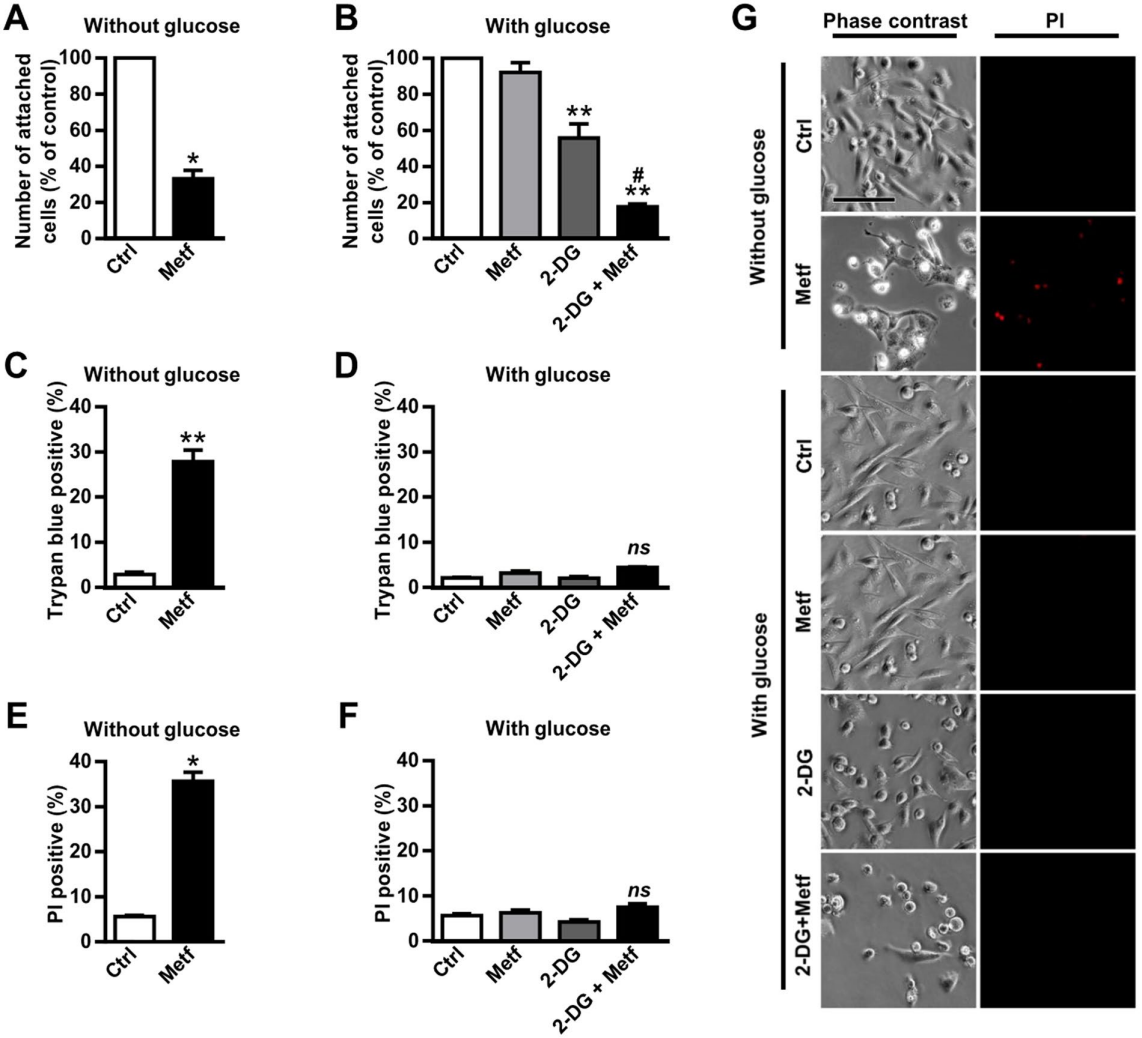
Combined treatment with metformin and 2-DG induces detachment of MDA-MB-231 cells. (**A**,**B**) MDA-MB-231 cells were grown for three days in medium without glucose containing 5 mM metformin (**A**) or in medium with (5.6 mM) glucose containing 5 mM metformin and/or 600 µM 2-DG (**B**). Number of attached cells was determined by Hoechst staining. Results are means ± SEM (n = 3). (**A**) *P = *0*.*0002* vs. Ctrl. (**B**) **P < *0*.*0001* vs. Ctrl; *#* = *0.045*, ^*#*^indicates synergism between metformin and 2-DG (see Supplementary Fig. S1). (**C**,**D**) MDA-MB-231 cells were grown for three days in medium without glucose and treated with 5 mM metformin (**C**) or in medium with (5.6 m M) glucose and treated with 5 mM metformin and/or 600 µM 2-DG (**D**). Cell survival was determined by Trypan blue staining. Results are means ± SEM (n = 4). **P < *0*.*0001* vs. Ctrl. (**E**,**F**) MDA-MB-231 cells were grown for three days in medium without glucose and treated with 5 mM metformin (**E**) or in medium with (5.6 mM) glucose and treated with 5 mM metformin and/or 600 µM 2-DG (**F**). Cell survival was determined by propidium iodide staining using flow cytometry. Results are means ± SEM (n = 3). *P = *0*.*0001* vs. Ctrl. (**G**) MDA-MB-231 cells were grown for three days in medium with (5.6 mM) or without glucose containing 5 mM metformin and/or 600 µM 2-DG. Cells were stained with propidium iodide and cell morphology and propidium iodide positive cells were observed with fluorescence microscopy. Scale bar corresponds to 20 μm.

The anti-proliferative effects of metformin on MDA-MB-231 cells depend on glucose availability in cell culture medium^47–50^. To mimic glucose concentrations in human serum^51^ or glucose depletion in the tumour core^52^, we performed most of the experiments on MDA-MB-231 cells in the presence of 5.6 mM glucose (medium with glucose) or in the absence of glucose (medium without glucose), respectively. We renewed medium every day to maintain well-defined glucose concentrations^48^. Consistent with previous studies^48, 49^, metformin reduced the number of attached cells in the medium without glucose (Fig. 1A). To inhibit glycolysis in the medium with glucose (5.6 mM), we used 600 µM 2-deoxy glucose (2-DG), a concentration that can be achieved in human serum after oral administration of 2-DG^36^. In the medium with glucose 5 mM metformin did not significantly alter the number of attached cells, while 2-DG reduced their number to 56%. Co-treatment with both compounds synergistically reduced the number of attached cells to 18% (Fig. 1B, Supplementary Fig. S1).

Since a combination of 5 mM metformin and 600 µM 2-DG in the medium with glucose or metformin in the medium without glucose profoundly decreased the number of attached cells, we expected reduced survival of MDA-MB-231 cells under both conditions. Thus, we determined the fraction of dead cells in the total cell population, which included floating as well as attached cells (Fig. 1C–F, Supplementary Fig. S2). In the absence of glucose, metformin increased the fraction of dead MDA-MB-231 cells to 28% (Fig. 1C) and 36% (Fig. 1E) of the total cell population, as determined by trypan blue and propidium iodide assay, respectively. In the medium with 5.6 mM glucose, metformin or 2-DG alone did not increase the fraction of dead MDA-MB-231 cells (Fig. 1D,F). Surprisingly, 96% and 92% of MDA-MB-231 cells remained viable also after combined treatment with metformin and 2-DG, as determined by trypan blue and propidium iodide assay, respectively (Fig. 1D,F).

Interestingly, numerous MDA-MB-231 cells treated for three days with metformin in the medium without glucose or with metformin and 2-DG in medium with 5.6 mM glucose were floating, i. e. they were round-shaped and detached from the bottom of the cell culture plate (Fig. 1G). Morphological alterations of the attached cells depended on the treatment. During co-treatment with metformin and 2-DG in the presence of 5.6 mM glucose MDA-MB-231 cells became round-shaped and then started to detach. Conversely, during treatment with metformin in the medium without glucose MDA-MB-231 cells became star-shaped cells before detachment. Using fluorescent microscopy and propidium iodide staining we additionally confirmed that combined treatment with metformin and 2-DG in medium with glucose induced detachment of cells that were viable. In contrast, many MDA-MB-231 cells treated with metformin in the absence of glucose were propidium iodide-positive (Fig. 1G).

### The majority of floating MDA-MB-231 cells obtained after co-treatment with metformin and 2-DG remain viable – Analysis of the attached and floating populations

**Figure 2:**
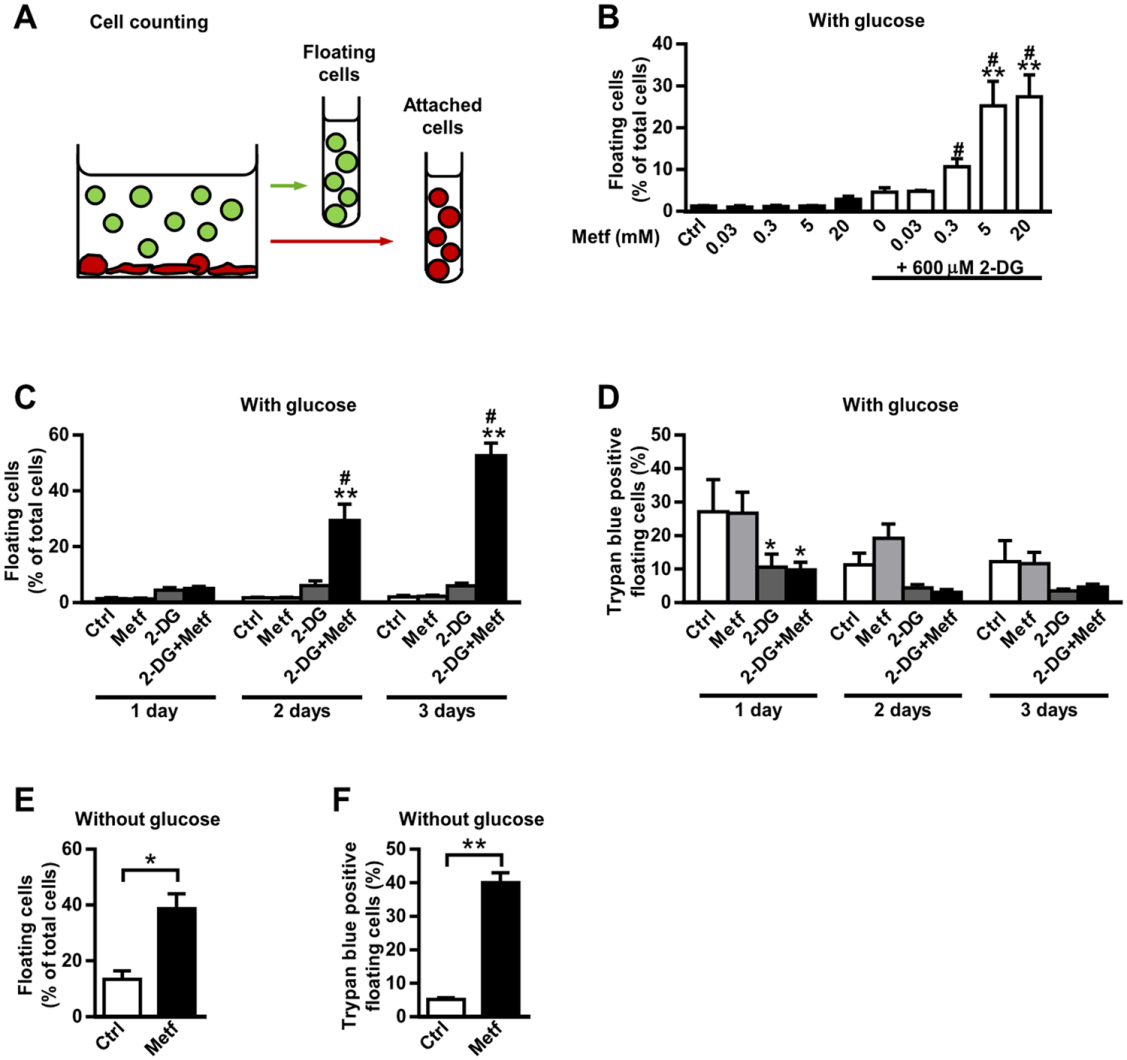
The majority of the floating MDA-MB-231 cells, obtained after co-treatment with metformin and 2-DG, remain viable – Analysis of the attached and floating populations. (**A**) Schematic representation of separation of MDA-MB-231 cells on two populations: floating cells (green) and attached cells (red). Floating cells were aspirated with a pipette and the remaining cells were regarded as attached cells. (**B**,**C**,**D**) MDA-MB-231 cells were grown for two days (**B**) or one to three days (**C**,**D**) in medium with (5.6 mM) glucose containing indicated concentrations of metformin and/or 600 µM 2-DG. The fraction of floating cells in each sample (**B**,**C**) and fraction of trypan blue positive cells in floating cell population (**D**) was determined using Countess cell counter. Results are means ± SEM (n = 3-4). *P ≤ *0*.*05* vs. Ctrl; **P < *0.0001* vs. Ctrl; ^#^indicates synergism between metformin and 2-DG (see Supplementary Figs S3 and S4). (**E**,**F**) MDA-MB-231 cells were grown for three days in medium without glucose containing 5 mM metformin. The fraction of floating cells in each sample (**E**) and fraction of trypan blue positive cells in floating cell population (**F**) was determined using Countess cell counter. Results are means ± SEM (n = 4). *P = *0.006*; **P < *0.0001*.

To determine the fraction of floating cells relative to the total cellular population, we separately collected and counted floating and attached cells (Fig. 2A). In the medium with glucose, metformin alone did not induce detachment of MDA-MB-231 cells (Fig. 2B,C). 2-DG alone tended to increase the percentage of the floating cells, but the difference was not statistically significant. In contrast, combined treatment with metformin and 2-DG caused massive, synergistic detachment of MDA-MB-231 cells in the medium with glucose (Fig. 2B,C, Supplementary Figs S3 and S4), which is in agreement with changes in cellular morphology (Fig. 1G). Detachment of MDA-MB-231 cells induced by 600 µM 2-DG and 5 mM metformin was dose and time-dependent (Fig. 2B,C).

We also determined the fraction of trypan blue-positive cells in the floating cell population (Fig. 2D). Importantly, after three-day treatment with 2-DG and metformin in the presence of glucose, 53% of MDA-MB-231 cells were floating (Fig. 2C), but only 5% of the floating cells were trypan blue-positive (Fig. 2D). In medium without glucose, three-day treatment with metformin increased fraction of floating cells to 39% (Fig. 2E). However, unlike during treatment with metformin and 2-DG in the presence of glucose, 40% of the floating cells were trypan-blue positive (Fig. 2F).

### Combined treatment with 2-DG and metformin suppresses cell proliferation

**Figure 3:**
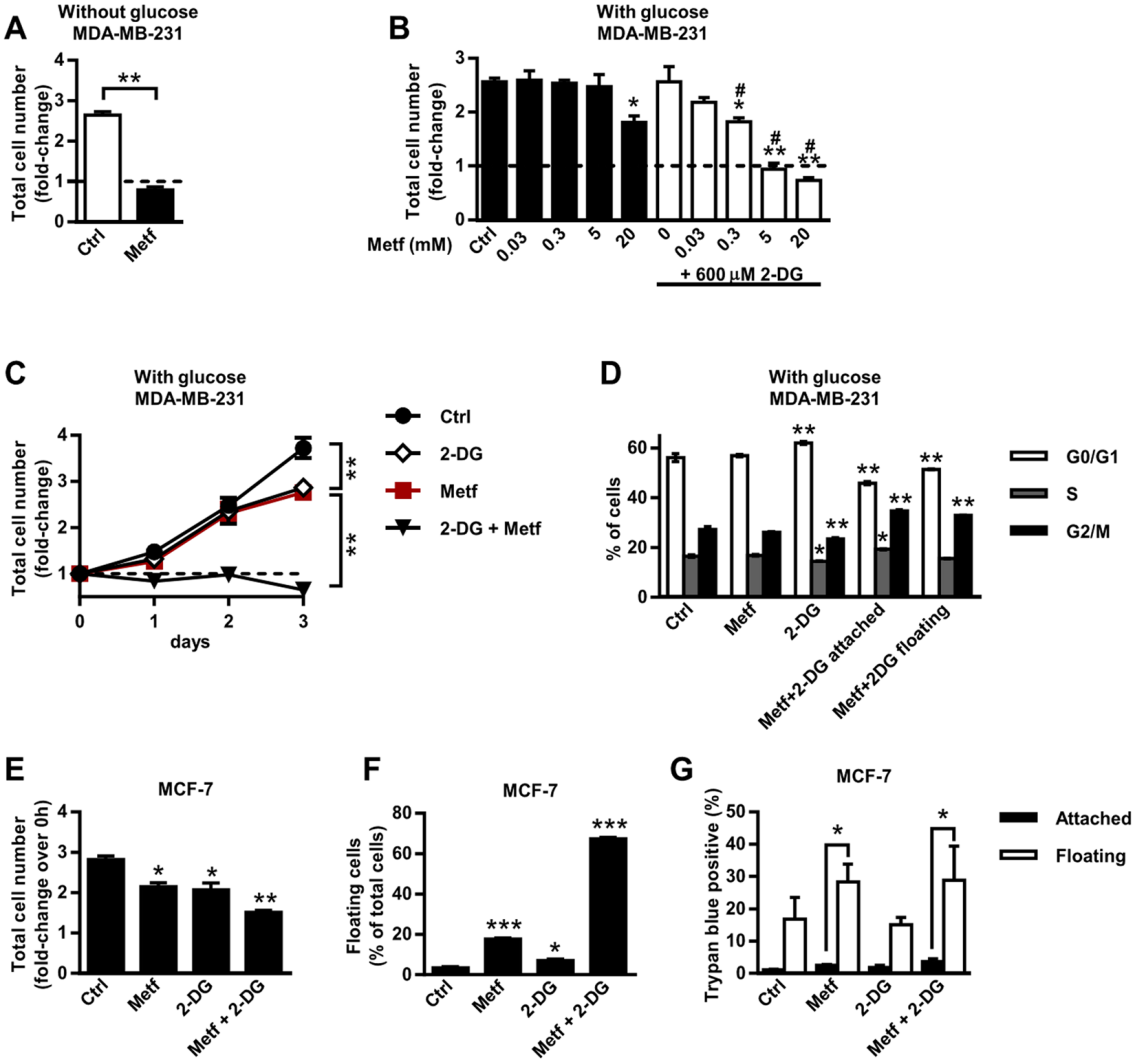
Combined treatment with 2-DG and metformin suppresses cell proliferation. (**A**) MDA-MB-231 cells were grown for three days in medium without glucose containing 5 mM metformin. Total number of cells in each sample was determined and normalized to the number of initially seeded cells. Results are means ± SEM (n = 4). **P < *0*.*0001*. (**B,C**) MDA-MB-231 cells were grown for two days (**B**) or one to three days (**C**) in medium with (5.6 mM) glucose containing indicated concentrations of metformin and/or 600 µM 2-DG. Total number of cells in each sample was determined and normalized to the number of initially seeded cells. Results are means ± SEM (n = 3–4). *P ≤ *0*.*05*; **P < *0*.*0001*. ^#^Indicates synergism between metformin and 2-DG (see Supplementary Fig. S5). (**D**) MDA-MB-231 cells were treated with metformin and 2-DG for two days. Cell cycle was analysed using flow cytometry. Results are means ± SEM (n = 2). *P ≤ *0*.*05* vs. Ctrl; **P < *0*.*0001* vs. Ctrl. (**E**,**F**,**G**) MCF-7 cells were treated with metformin and 2-DG for three days and their effects on total cell number (**E**), percentage of floating cells (**F**) and trypan blue positive cells (**G**) were analysed. Results are means ± SEM (n = 3).

Metformin suppressed proliferation of MDA-MB-231 cells in the medium without glucose (Fig. 3A). Combined treatment with 600 µM 2-DG and 0.3–20 mM metformin in the medium with glucose also suppressed their proliferation after a two-day treatment (Fig. 3B). Furthermore, 5 mM metformin or 2-DG alone slightly suppressed proliferation of MDA-MB-231 cells after a three-day treatment (Fig. 3C), whereas only 20 mM metformin significantly suppressed proliferation after a two-day treatment (Fig. 3B). Even though a three-day treatment with 2-DG reduced the number of the attached cells (Fig. 1B) and total number of cells (Fig. 3C), most of the effect was probably due to reduced proliferation, since only very small fraction of the floating cells was observed (Fig. 2B,C). Since the total number of cells was reduced after a three-day treatment with both metformin and 2-DG (Fig. 3C), we performed a cell cycle experiment after a two-day treatment with both compounds.

To investigate the effect on the cell cycle, MDA-MB-231 cells were treated with 2-DG and metformin for two days and then DNA content was measured with flow cytometry. Co-treatment with 2-DG and metformin increased population of cells in G2/M phase, indicating arrest in G2 and/or mitotic (M) phase of the cell cycle (Fig. 3D, Supplementary Fig. S6). This effect was observed in both attached and floating populations of cells.

To determine whether the effects of combined treatment with metformin and 2-DG on cell detachment are cell-line specific, we examined them in MCF-7 breast cancer cells (Fig. 3E–G) and PC-3 prostate cancer cells (Supplementary Fig. S7). MCF-7 cells express estrogen receptor and progesterone receptor, but are negative for human epidermal growth factor receptor 2 (HER-2)^53^, while PC-3 cells are highly metastatic and negative for androgen receptor and prostate-specific antigen^54^. Metformin and/or 2-DG suppressed proliferation of MCF-7 cells after a three-day treatment (Fig. 3E). Metformin and 2-DG alone increased the percentage of floating MCF-7 cells to 18% and 7%, respectively, whereas combined treatment with both compounds increased their percentage to about 67% (Fig. 3F). 2-DG alone did not significantly increase the percentage of dead floating MCF-7 cells. Metformin alone and combined with 2-DG increased the percentage of dead floating MCF-7 cells to about 28% and 29%, respectively (Fig. 3G). In contrast, combined treatment of PC-3 cells with 2-DG and metformin reduced total number of cells, but did not increase the percentage of floating or dead cells (Supplementary Fig. S7).

### Floating MDA-MB-231 cells obtained after combined treatment with metformin and 2-DG proliferate upon re-seeding

**Figure 4:**
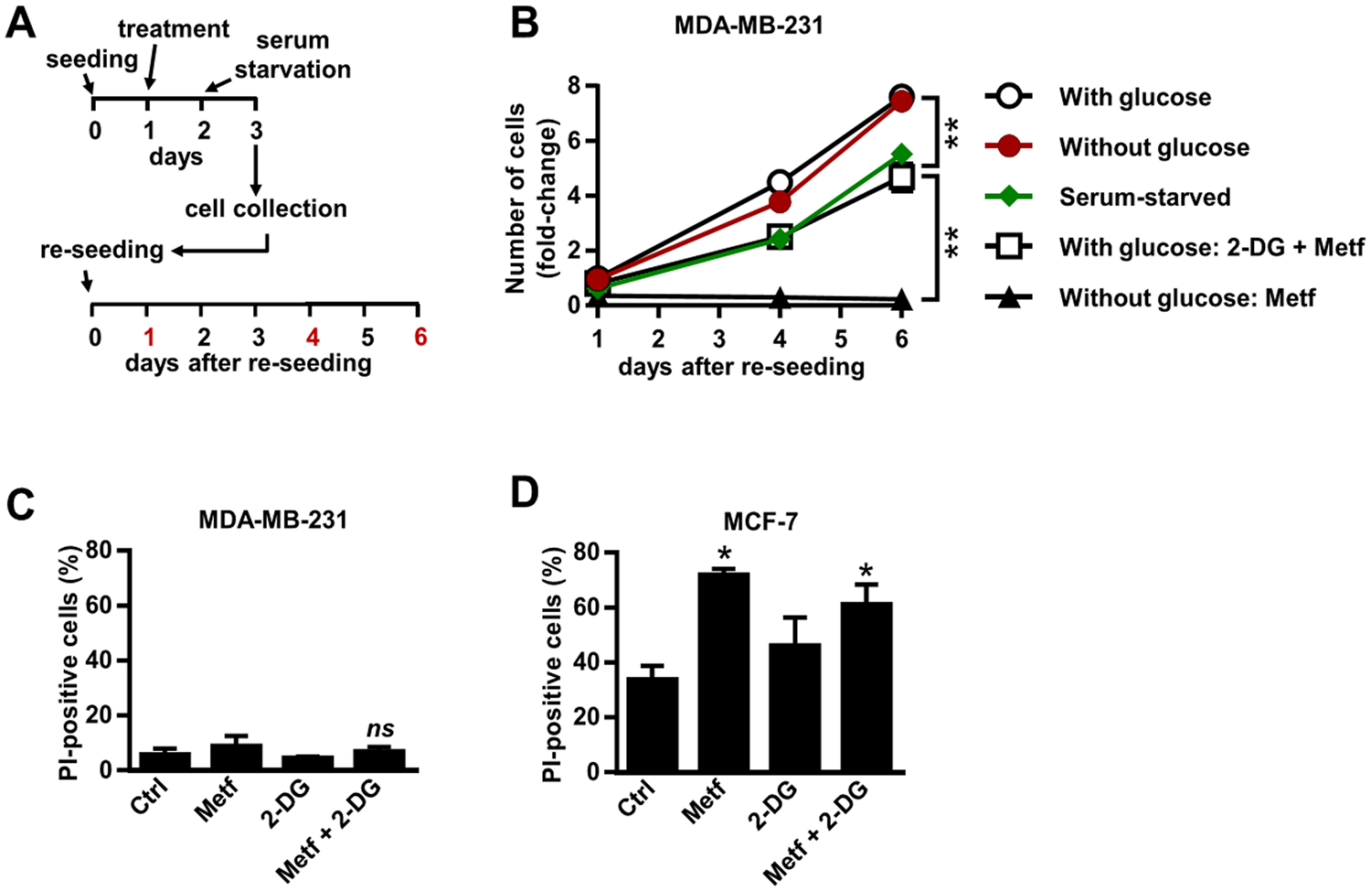
Floating MDA-MB-231 cells obtained after combined treatment with metformin and 2-DG proliferate upon re-seeding. (**A,B**) MDA-MB-231 cells were treated with 5 mM metformin in medium without glucose for two days. Alternatively, MDA-MB-231 cells were cultured in medium with (5.6 mM) glucose containing 5 mM metformin and/or 600 µM 2-DG for two days. Only floating cells were collected, washed and resuspended in a standard pharmacological compound-free cell culture medium containing high, 25 mM glucose. Similarly, attached MDA-MB-231 cells grown in medium with (5.6 mM) or without glucose for two days or in the absence of serum for one day were collected, washed and resuspended in a standard pharmacological compound-free culture medium containing high, 25 mM glucose. Then, 20.000 of MDA-MB-231 cells from each group were reseeded in 24 well plates. Relative number of cells was determined one, four and six days after re-seeding by Hoechst assay. Results are means ± SEM (n = 3). **P < *0*.*0001*. (**C**,**D**) MDA-MB-231 cells (**C**) and MCF-7 cells (**D**) were treated for three days with 5 mM metformin and 600 µM 2-DG in suspension using cell culture plates covered with poly-HEMA, which prevents cell adhesion. The percentage of propidium iodide-positive cells was determined using flow cytometry. Results are means ± SEM (n = 3). *P ≤ *0*.*05* vs. Ctrl.

Results obtained with propidium iodide and trypan blue assay suggest that floating MDA-MB-231 cells, obtained after combined treatment with metformin and 2-DG in medium with glucose are mostly viable (Figs 1 and 2). To further confirm this observation and to analyse their proliferation potential, we reseeded floating MDA-MB-231 cells obtained after two-day incubation with (i) metformin and 2-DG in the medium with 5.6 mM glucose and (ii) metformin alone in medium without glucose. As non-treated controls, we used MDA-MB-231 cells that were attached and grown for two days (iii) in the medium without glucose or (iv) in the medium with 5.6 mM glucose before reseeding. In all cases, MDA-MB-231 cells were reseeded in the standard cell culture medium containing high concentration (25 mM) of glucose and supplemented with 10% FBS (Fig. 4A,B). We determined the relative number of cells one, four and six days after reseeding.

Non-treated control MDA-MB-231 cells that were attached and grown in the medium with 5.6 mM glucose and without glucose before reseeding had similar proliferation rate (Fig. 4B). Floating cells that were collected after two-day treatment with metformin in the medium without glucose were unable to proliferate after reseeding. In contrast, the floating cells that were collected after combined treatment with metformin and 2-DG (medium with glucose) were proliferating after reseeding. Since treatments that increased the fraction of floating cells also suppressed proliferation of MDA-MB-231 cells (Figs 2B,C and 3B,C), we used serum starvation, which is commonly used to attenuate cellular proliferation, as another control^55^. Hence, we did a parallel experiment, in which we re-seeded cells that were attached and serum-starved for 24 hours. MDA-MB-231 cells that had been serum-starved proliferated with the same rate as the cells that were viable and floating before reseeding but had lower proliferation rate than the cells in non-treated controls (Fig. 4B).

We re-seeded MDA-MB-231 cells after a two-day treatment, since the number of floating cells in this period of time was sufficient to perform the experiment (Fig. 4B). Furthermore, this time point was used to study the effects of combined treatment with metformin and 2-DG on cells that detached, but were not exposed to both compounds in a suspension for an additional period of time. To evaluate if metformin and 2-DG induce anoikis, we cultured MDA-MB-231 and MCF-7 cells in the absence or presence of 5 mM metformin and/or 600 µM 2-DG for three days on cell culture plates covered with poly-HEMA, which prevents cell attachment (Fig. 4C,D). Untreated MDA-MB-231 cells were resistant to anoikis, consistent with previous studies^7^. 2-DG and/or metformin did not increase the percentage of dead MDA-MB-231 cells, as determined by propidium iodide assay using flow cytometry (Fig. 4C). In contrast, about 34% of MCF-7 untreated cells were dead when grown in an anchorage-independent condition (Fig. 4D). Metformin and 2-DG alone increased the percentage of dead MCF-7 cells to about 72% and 46%, respectively, while combined treatment (metformin and 2-DG) increased the number of dead MCF-7 cells to about 61% (Fig. 4D).

### AMPK activation is needed for detachment of viable MDA-MB-231 cells

**Figure 5:**
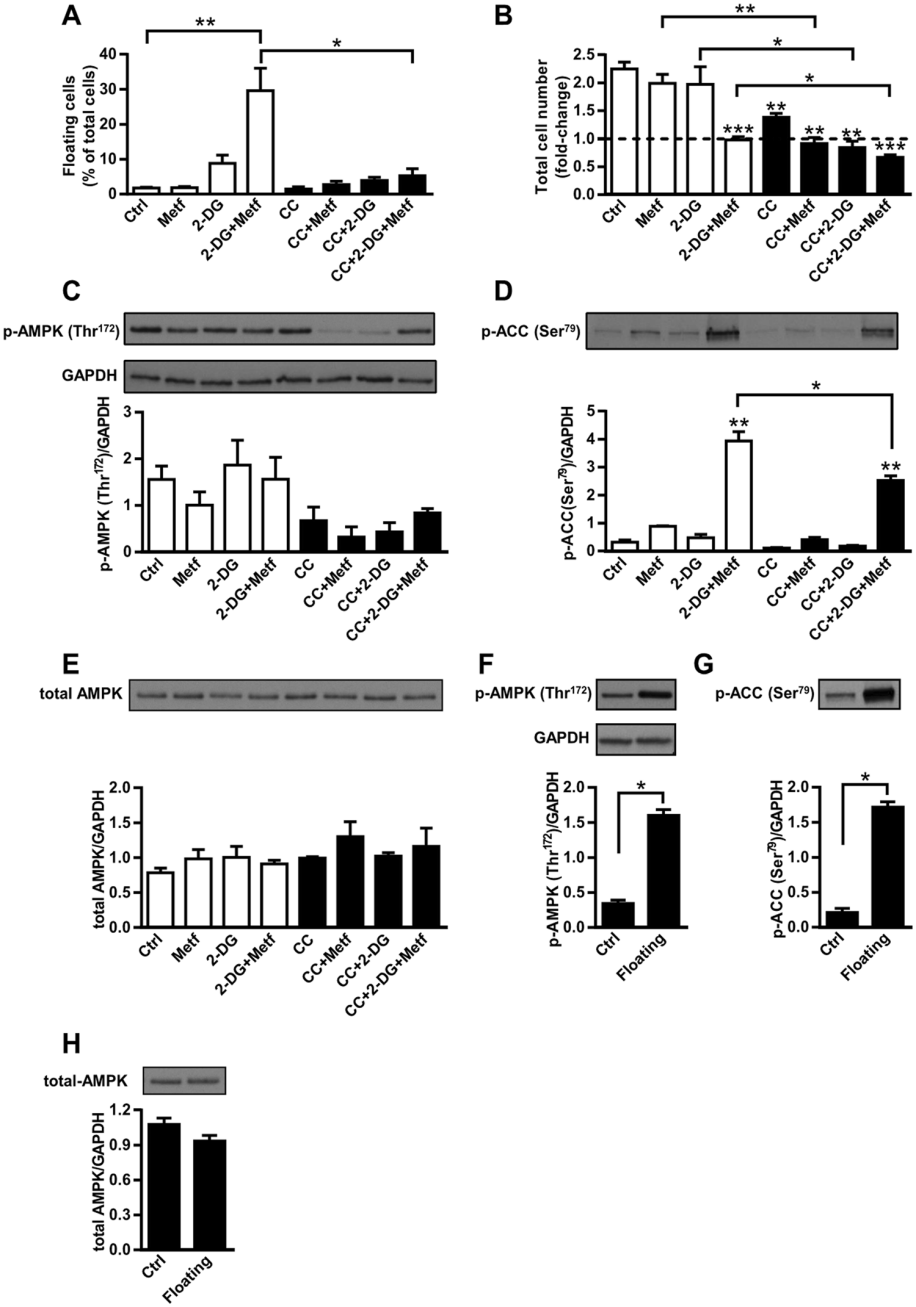
AMPK activation is needed for detachment of viable MDA-MB-231 cells. (**A**,**B**) MDA-MB-231 cells were pre-treated for 2 h with 5 µM Compound C in medium with glucose. Then 5 mM metformin and/or 600 µM 2-DG were added. After two days, the fraction of floating cells in each sample (**A**) and total number of cells normalized to the number of seeded cells (**B**) were determined. Results are means ± SEM (n = 3). (**A**)*P ≤ *0*.*001*; **P < *0*.*0001*. **(B**) *P ≤ *0*.*05*; **P ≤ *0*.01; ***P < *0*.*001*. (**C–E**) MDA-MB-231 cells were pre-treated with 5 µM Compound C in medium with glucose. Then 5 mM metformin and 600 µM 2-DG were added for additional 24 h. Phosphorylation of AMPK (**C**) and ACC (**D**) and total AMPK level **(E**) were determined by Western blot analysis. Cropped blots are shown. Uncropped blots are presented in Supplementary Fig. S9. Results are means ± SEM (n = 3). *P ≤ *0*.*05*; **P < *0*.*0001*. (**F**,**G**,**H**) MDA-MB-231 cells were grown in a regular cell culture plate or in a cell culture plate covered with poly-HEMA (to prevent cell adhesion) for three days. Phosphorylation of AMPK (**F**), ACC (**G**) and total AMPK level (**H**) were determined by Western blot analysis. Cropped blots are shown. Uncropped blots are presented in Supplementary Fig. S9. Results are means ± SEM (n = 3). *P < *0*.*0001*.

Combination of metformin and 2-DG synergistically activates AMPK^44, 46^. AMPK activation helps cancer cells to survive in an anchorage-independent condition^15^. We therefore speculated that AMPK activation might be correlated with detachment of viable MDA-MB-231 cells. To block AMPK activation we used 5 µM Compound C, which markedly suppressed detachment of MDA-MB-231 cells incubated with 5 mM metformin and 600 µM 2-DG (Fig. 5A). Compound C alone markedly reduced proliferation of MDA-MB-231 cells, indicating it does not only inhibit AMPK but probably also affects other cell functions. Combined treatment with Compound C, metformin and 2-DG decreased the number of MDA-MB-231 cells in comparison to control (untreated) MDA-MB-231 cells (Fig. 5B). Furthermore, Compound C further reduced the number of cells treated with metformin and/or 2-DG in comparison to the same treatment without Compound C. To exclude that Compound C prevented detachment of MDA-MB-231 cells due to DMSO in which we prepared Compound C, we co-treated MDA-MB-231 cells for three days in the presence of 0.1% (final concentration of DMSO used in our experiments) and 1% DMSO with combination of 600 µM 2-DG and 5 mM metformin (Supplementary Fig S8). Neither 1% nor 0.1% DMSO prevented cell detachment induced by the combined treatment with metformin and 2-DG.

Using Western blot we determined the effect of 5 mM metformin, 600 µM 2-DG and 5 µM Compound C on the phosphorylation of AMPK (Fig. 5C, Supplementary Fig. S9) and its direct target ACC (Fig. 5D, Supplementary Fig. S9). Metformin or 2-DG alone did not alter phosphorylation of AMPK, whereas metformin tended to increase phosphorylation of ACC. Combined treatment with metformin and 2-DG did not alter phosphorylation of AMPK (Fig. 5C), but markedly increased phosphorylation of ACC (Fig. 5D). Compound C reduced phosphorylation of ACC in MDA-MB-231 cells co-treated with 2-DG and metformin (Fig. 5D). These treatments did not affect total level of AMPK (Fig. 5E). To determine the basal AMPK phosphorylation in untreated floating cells, we cultured MDA-MB-231 cells on cell culture plates covered with poly-HEMA, which prevents cell attachment. The floating cells had markedly higher phosphorylation of AMPK as well as ACC than attached cells (Fig. 5F,G), whereas total AMPK level remained unaltered (Fig. 5H).

### AMPK activators AICAR and A-769662 do not induce detachment of viable MDA-MB-231 cells

**Figure 6:**
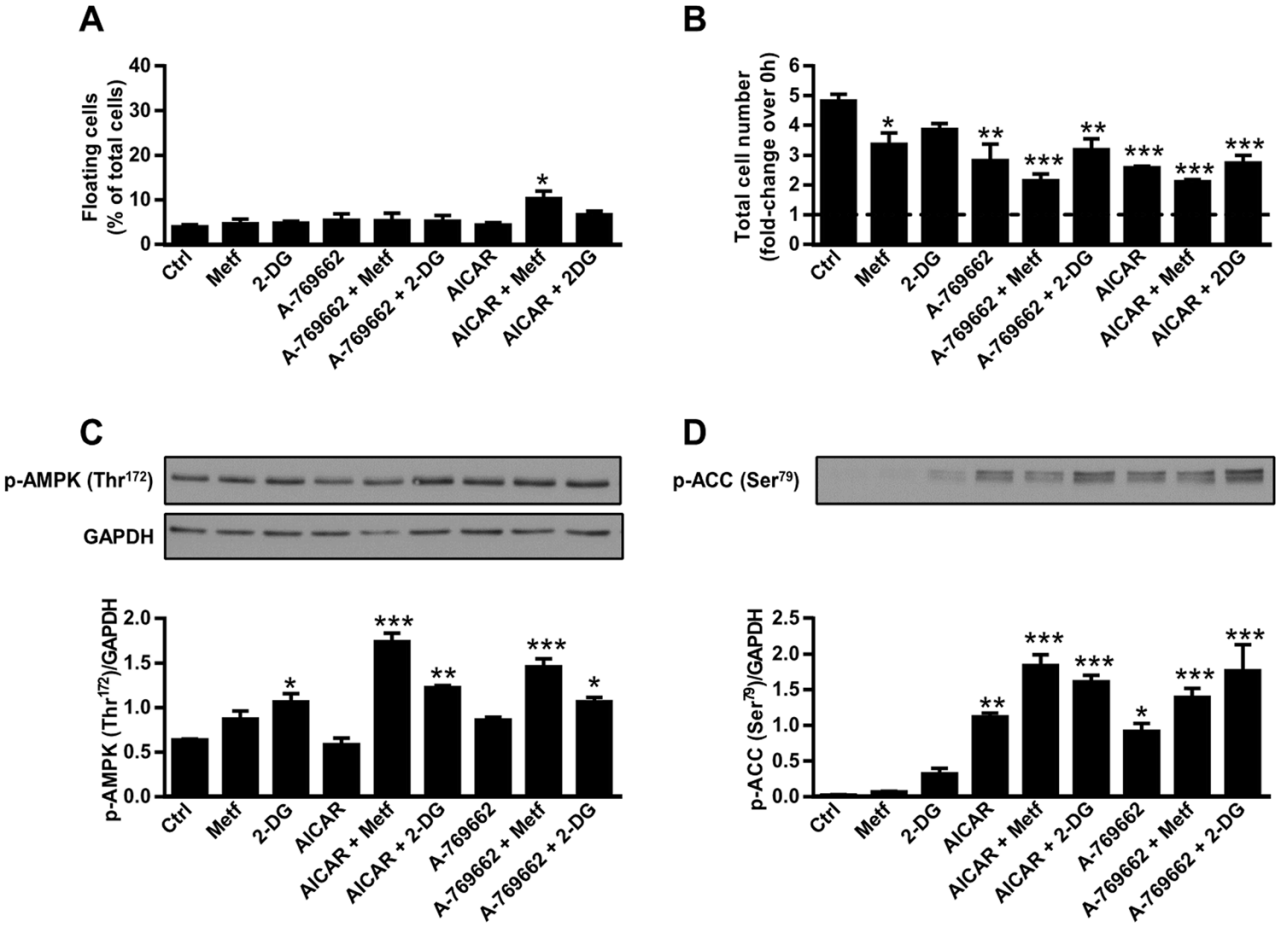
AMPK activators AICAR and A-769662 do not induce detachment of viable MDA-MB-231 cells. (**A**,**B**) MDA-MB-231 cells were cultured in the presence of 5 mM metformin, 600 µM 2-DG, 500 µM AICAR and 100 µM A-769662 and 5.6 mM glucose for three days. The percentage of floating cells (**A**) and total cell number (**B**) were determined using Countess cell counter. Results are means ± SEM (n = 3). (**C**,**D**) MDA-MB-231 cells were cultured in the presence of 5 mM metformin, 600 µM 2-DG, 500 µM AICAR and 100 µM A-769662 and 5.6 mM glucose and in the absence of serum for 24 h. Phosphorylation of ACC and AMPK were determined using Western blot. Cropped blots are shown. Uncropped blots are presented in Supplementary Fig. S10. Results are means ± SEM (n = 2). *P ≤ *0*.*05*; **P ≤ *0*.01; ***P < *0*.*001* vs. Ctrl.

To examine effects of direct AMPK activators on cell detachment, we treated MDA-MB-231 cells for three days with 500 µM AICAR and 100 µM A-769662 alone or in combination with 5 mM metformin or 600 µM 2-DG (Fig. 6A). Only combined treatment with AICAR and metformin slightly increased the percentage of floating cells, while their percentage was unaltered during treatment with AICAR or A-769662 alone or any other treatment combination (Fig. 6A). AICAR and A-769662 alone and combined with 2-DG or metformin significantly supressed proliferation of MDA-MB-231 cells (Fig. 6B).

To examine the effects of AICAR and A-769662 on AMPK activation, we treated MDA-MB-231 cells for 24 h with both compounds alone or combined with metformin and 2-DG (Fig. 6C,D, Supplementary Fig. S10). AICAR and A-769662 increased phosphorylation of AMPK only when combined with metformin or 2-DG (Fig. 6C). Nevertheless AICAR and A-769662 alone increased phosphorylation of ACC (Fig. 6D), indicating AMPK was probably activated allosterically by these treatments^56, 57^.

## Discussion

Detachment of cancer cells from extracellular matrix is an early step in the metastatic cascade^3^. Here we show that combined treatment with 5 mM metformin and 600 µM 2-DG completely blocks proliferation of MDA-MB-231 cells. However, combined treatment with both compounds also increased the fraction of floating cells up to about 53%, of which 95% were viable (Fig. 2C,D). Similarly, 2-DG and metformin increased percentage of floating MCF-7 cells, of which about 71% were viable (Fig. 3F,G). These observations have several important implications. Detachment of cancer cells in cell culture is often ignored, although it could be important for interpretation of results. Potential anti-cancer compounds are often analysed on a large scale with rapid proliferation tests, such as MTS, crystal violet, and Hoechst assay, which give the information about the relative number of cells that are attached to the bottom of the cell culture plate. Furthermore, only attached or total cell populations are usually analysed on flow cytometry, thus disregarding potential viability of floating cells. High throughput tests that analyse only attached or total cell populations might therefore miss physiologically relevant effects of some compounds, which can manifest in detachment of viable cancer cells.

Surprisingly, the combination of metformin and 2-DG induced time-dependent detachment of viable MDA-MB-231 cells *in vitro*. We therefore extended our study to prostate cancer PC-3 cells and breast cancer MCF-7 cells. Combined treatment with metformin and 2-DG did not increase the percentage of dead PC-3 cells; however, it also did not induce their detachment (Supplementary Fig. S7). Since PC-3 cells are p53-null^58^ and MDA-MB-231 cells have mutant p53^5^, our results in terms of cell viability are broadly consistent with the study of Ben Sahra *et al*., who demonstrated that the combination of 2-DG and metformin does not reduce viability of prostate cancer cells deficient or mutated for p53^46^. In our study, co-treatment with metformin and 2-DG of MCF-7 cells, which express wild type p53, increased the percentage of floating cells to about 67%, of which about 29% were dead (Fig. 3F,G). MCF-7 cells are less resistant to an anchorage-independent condition than MDA-MB-231 cells (Fig. 4C,D). Since attached MCF-7 cells were viable after combined treatment with metformin and 2-DG (Fig. 3G), it is possible that MCF-7 cells died due to anoikis, which was further promoted by the energy stress induced by metformin and 2-DG (Fig. 4D), and not due to the detachment process itself. Taken together, these results indicate that the response of various cancer cell lines to metformin and 2-DG treatment depends on cell line characteristics.

Compared with other cell lines used in this and in other studies^59^, MDA-MB-231 cells poorly responded to metformin in medium with glucose. This is consistent with studies showing that MDA-MB-231 cells are resistant to millimolar metformin concentrations if glucose availability in cell culture medium is sufficient^48–50, 60, 61^. In our study, metformin did not increase phosphorylation of AMPK, while it tended to increase phosphorylation of its downstream target ACC (Figs. 5C,D and 6C,D). Conversely, several studies reported that metformin activates AMPK in MDA-MB-231 cells^50, 62^. Possible reasons for this apparent discrepancy include variations in glucose availability in cell culture media^48–50^, medium renewal protocol^48^, type of cell culture medium used^63^, incubation time^62^ and specific properties of cell lines used^29^. Variation in these parameters might also explain why some studies suggest that the combination of metformin and 2-DG reduces viability of various cancer cell lines, independent of their p53 status^44, 45^ as well as why sensitivity of MDA-MB-231 cells to metformin differs between studies^48, 49, 59^. For example, cancer cells consume large amounts of glucose as an energy source and consequently glucose is gradually depleted from the cell culture medium^29, 48^. Consistent with our results, glucose depletion markedly increases the toxicity of metformin for various cancer cells^29, 47–50^. Metformin treatment attenuates the flow of metabolic intermediates into the Krebs cycle^33^. Metformin-treated cells must consequently increase the rate of glycolysis to balance their energetic demands^49^, which is not possible in the absence of glucose. Therefore, metformin-treated cells cannot satisfy their energetic demands in the absence of glucose, which might explain their reduced proliferation rate, changes in cell shape and ultimately cell death. Since glucose depletion can interfere with the results when metformin is used, we tightly controlled glucose concentration in all our experiments with daily medium renewal^48^.

The floating MDA-MB-231 cells, obtained after combined treatment with 2-DG and metformin were able to proliferate after reseeding in a fresh compound-free cell culture medium, which again shows that they are viable. Notably, the floating viable MDA-MB-231 cells were in the supernatant for as long as two days before they were resuspended in a fresh cell culture medium and allowed to reattach. Therefore, the floating viable cells were not only resistant to anoikis but were also able to reattach and divide after reseeding (Fig. 4B). Combined treatment with metformin and 2-DG induced detachment of MCF-7 (Fig. 3F); however, these cells died after three days in an anchorage-independent condition (Fig. 4D). These results clearly demonstrate the difference in two important steps in metastasis formation: detachment of cancer cells and their survival in an anchorage-independent condition^3^. Importantly, cancer cells *in vivo* are probably continuously detaching from extracellular matrix and travelling to distant organs^64^, but only a small fraction survives in a lymph or/and blood stream and an even smaller fraction ultimately colonizes distant organs. Combined treatment with 2-DG and 5 mM metformin did not only increase the number of floating cells, but also suppressed proliferation of MCF-7 cells (Fig. 3E) and MDA-MB-231 cells (Fig. 3B–D). These results indicate that suppression of proliferation and cell detachment might be linked, consistent with studies which showed that attenuated proliferation and increased metastatic rate of cancer cells are correlated^11, 65–68^.

AMPK activation was markedly higher in floating than in attached MDA-MB-231 cells (Fig. 5F,G). Since AMPK is involved in anoikis resistance^15, 17^, it could also play a role in detachment of MDA-MB-231 cells. Consistent with previous studies^44–46^, our results show that the combination of 2-DG and metformin synergistically activates AMPK, which was observed as an increase in phosphorylation of ACC (Fig. 5D). Notably, Compound C, an AMPK inhibitor, reduced phosphorylation of ACC and prevented detachment of MDA-MB-231 cells treated with metformin and 2-DG (Fig. 5A,D). Thus our results suggest that AMPK activation is necessary for detachment of viable MDA-MB-231 cells after combined treatment with 2-DG and metformin. However, direct AMPK activators AICAR and A-769662 did not increase the number of viable floating MDA-MB-231 cells (Fig. 6A), indicating AMPK activation per se is not sufficient to trigger detachment of viable cells.

Multiple studies demonstrated a pro-survival role for AMPK activation for cells in energetic crisis^69^ and in an anchorage-independent condition^15, 17^. Under these conditions, AMPK activation prevents cell death, because it increases cellular catabolic processes, such as fatty acid beta oxidation, and consequently balances cellular energy and redox homeostasis^15^. However, both 2-DG and metformin also have AMPK-independent effects on cells^33, 70^ that may induce detachment without activating AMPK. 2-DG increases metabolic flux through pentose phosphate pathway^15, 41, 42^, which contributes to increased ratio of NADPH/NADP and reduced level of reactive oxygen species, preventing anoikis in an AMPK-independent manner^15^. Moreover, recent study demonstrated that anchorage-independent condition *per se* induces reductive carboxylation in various cancer cells^16^. Interestingly, reductive glutamine metabolism is also induced by metformin^32–34^. Although AMPK activation alone is not a sufficient signal for cell detachment, our results show that it is an important step in MDA-MB-231 cell detachment process.

In conclusion, here we show that co-treatment with metformin and 2-DG suppresses proliferation and triggers detachment of MDA-MB-231 cells *in vitro*. Furthermore, about 95% of the floating MDA-MB-231 cells were viable and able to proliferate upon re-seeding in a pharmacological compound-free cell culture medium. Similar effect of combined treatment with metformin and 2-DG on detachment was observed on MCF-7 cells; however, MCF-7 cells were less resistant to anoikis. Our results indicate that AMPK activation is a necessary step but alone not a sufficient trigger for detachment of MDA-MB-231 cells, since neither AICAR nor A-769662 in combination with metformin or 2-DG had any major effect on the percentage of floating cells. Taken together, our results show that detachment is not a reliable indicator of death in cultured cancer cells. Survival of floating cancer cells should therefore be taken into account during assessment of potential anti-cancer compounds in cell culture.

## Materials and Methods

### Antibodies and reagents

Antibodies against GAPDH (sc-25778) were purchased from Santa Cruz Biotechnology. Antibodies against phospho-ACC (Ser^79^) (CST3661), phospho-AMPKα (Thr^172^) (CST2535) and total AMPK (CST2532) were purchased from Cell Signaling Technology. Bis-Tris 4–12% polyacrylamide gels (345–0123), MES running buffer (161–0789) and horseradish peroxidase secondary antibody conjugate (170–6515) were obtained from Bio-Rad. Polyvinylidene Fluoride (PVDF) Immobilon-P membrane (IPVH00010) was manufactured by Merck Millipore. Protein molecular weight marker (RPN800E) was purchased from GE Healthcare Life Sciences, while enhanced chemiluminescence (ECL) reagent (32209) and BCA protein assay were purchased from Life Technologies. Metformin was obtained from Calbiochem (Merck Millipore), 2-deoxy-D-glucose from Sigma-Aldrich and Compound C (Dorsomorphin) and A-769662 from Abcam. All other reagents, unless otherwise specified, were purchased from Sigma-Aldrich or Merck Millipore.

### Cell culture

MDA-MB-231, MCF-7 and PC-3 cancer cell lines were obtained from ATCC (USA). MDA-MB-231 cells and PC-3 cells were routinely grown in RPMI-1640 medium (Genaxxon bioscience, Germany) without pyruvate, supplemented with 2 mM L-glutamine (Sigma-Aldrich Co.), 25 mM glucose (4.5 g/L of glucose; high glucose concentration) (Sigma-Aldrich) and 10% fetal bovine serum (FBS; Sigma-Aldrich) in a humidified atmosphere at 37 °C, 5% CO_2_. If not stated otherwise, all experiments were performed in RPMI-1640 medium supplemented with 2 mM L-glutamine, 10% FBS and 0 mM (medium without glucose) or 5.6 mM glucose (1 g/L of glucose; medium with glucose). MCF-7 cells were grown in Eagle’s Minimum Essential Medium (Sigma, M5650), containing 5.6 mM glucose and supplemented with 2 mM L-glutamin and 10% FBS, in a humidified atmosphere at 37 °C, 5% CO_2_. To control glucose concentration in the culture medium, all experiments were performed with daily medium renewal^48^, unless stated otherwise.

### Analysis of the number of total, floating and Trypan-blue positive cells

Cells were seeded in a six well plate. Next day, cells were washed twice with phosphate buffered saline (PBS) and placed in a fresh medium with 0 mM (medium without glucose) or 5.6 mM glucose (medium with glucose) with treatments. Metformin, 2-DG and AICAR were dissolved in PBS to prepare 1 M, 60 mM and 250 mM stock solution, respectively. Compound C and A-769662 were dissolved in DMSO to obtain 5 mM and 100 mM stock solution, respectively. If needed, stock solutions were appropriately diluted before addition to cell culture medium, so the final concentration of PBS and DMSO in cell culture medium was 1% and 0.1%, respectively. As a vehicle control, DMSO and/or PBS were added to un-treated cells to achieve 0.1% and 1% final concentrations, respectively. If Compound C was used, MDA-MB-231 cells were pre-treated for 2 hours, before addition of other compounds. On the day of the experiment, supernatants were collected for determination of the number of floating cells. The remaining adherent cells in cell culture wells were trypsinised and separately collected for determination of the number of attached cells. Floating and attached cells were then centrifuged at 290 rcf for 8 min and resuspended in a known volume of medium. Cells were stained with trypan blue and the number of attached (AC) and floating cells (FC) was counted separately, using a Countess automated cell counter (Invitrogen). In each population the number of trypan blue positive cells (TB) was also determined. Total number of cells was calculated as a sum of the floating and attached cells. Number of total cells was normalized to the number of seeded cells. The fraction of floating cells was calculated: %FC = 100 × FC/(FC + AC). The fraction of trypan blue positive (dead) cells in each sample was calculated as %TB_FC_ = 100 × TB_FC_/FC for the floating cells or %TB_AC_ = 100 × TB_AC_/AC for the attached cells. Similarly, the percentage of trypan blue positive cells in the total cellular population was determined as:

1 $$ \% {\rm{TB}}=100\times ({{\rm{TB}}}_{{\rm{FC}}}+{{\rm{TB}}}_{{\rm{AC}}})/({\rm{FC}}+{\rm{AC}}).$$

### Number of attached cells determined by Hoechst assay

Number of attached cells was determined by Hoechst assay as previously described^48^. Briefly, to determine the number of attached cells at different time points, cell supernatants were discarded and MDA-MB-231 cells were stored at −20 °C at each time point. When all the samples included in one experiment were collected, MDA-MB-231 cells were thawed and lysed with a 0.04% SDS solution at room temperature. Then, buffer containing 50 mM TRIS-HCl, 100 mM NaCl (pH = 8.25) and 5 µg/ml Hoechst 33342 stain (Thermo Fisher Scientific) was added to cell lysates. Relative quantity of released dsDNA in each sample was determined. Alternatively, if MDA-MB-231 cells were analysed only at one time point, non-lysed cells were stained with Hoechst 33342 for 30 minutes and washed. Fluorescence intensity in both cases was determined at 350 nm excitation and 461 nm emission using Tecan Infinite 200 (Tecan, Männedorf, Switzerland).

### Propidium iodide staining

Floating and adherent MDA-MB-231 cells were joined, centrifuged at 290 rcf for 8 min and resuspended in PBS. Propidium iodide was added (0.15 mM final concentration) and MDA-MB-231 cells were analysed with CyFlow space flow cytometer (Partec). Propidium iodide signal of about 1 × 10^4^ events per sample was collected using FL3 filter (675/25). The fraction of propidium iodide positive cells in each sample was determined using FlowJo software. Gates on histograms were set based on positive and negative control (Supplementary Fig. S2). As a positive control, we used MDA-MB-231 cells, which were grown in NaCl for two days. At least three independent experiments were performed.

To visually confirm results obtained by flow cytometry, propidium iodide positive cells were also observed using fluorescence microscope Olympus IX81. Propidium iodide was added directly to each cell culture well containing RPMI-1640 medium with treatments. Three independent experiments were performed and at least five representative images were taken. Images were not used for quantitative analysis.

### Cell cycle

For cell cycle analysis, MDA-MB-231 cells were seeded in six well culture plates. After 24 h cells were washed with PBS and placed in a fresh medium with 5.6 mM glucose and treatments. After two days, cells were harvested and centrifuged at 290 rcf for 8 min. Then the pellet was resuspended in 0.1% triton X-100 solution in PBS in the presence of propidium iodide and RNase (Ribonuclease A from bovine pancreas, Sigma Aldrich, Germany) and incubated in the dark for 20 minutes. Cells were analyzed for DNA content using Attune™ NxT flow cytometer (Thermo Fisher Scientific, Waltham, USA). The propidium iodide signal was collected using the BL-2 filter (574/26). Samples were prepared in duplicate and analyzed on at least 2 × 10^4^ events per sample. Analysis was performed with Attune® Cytometric Software (Thermo Fisher Scientific, Waltham, USA; see Supplementary Fig. S6).

### Anchorage-independent growth

Cell culture dishes (surface area was 22.1 cm^2^) were covered with warm poly(2-hydroxyethyl methacrylate) (Sigma-Aldrich, P3932; poly-HEMA) dissolved in 95% ethanol and left to dry at 37 °C^71^. MDA-MB-231 cells were seeded in a poly-HEMA-coated dish or a control dish and analysed with Western blotting.

To analyse anoikis resistance of cells treated with metformin and 2-DG, cells were seeded on a poly-HEMA-coated 12-well plates and treated with compounds in medium with 5.6 mM glucose for three days. Cells were analysed by propidium iodide staining, using Attune™ NxT Flow Cytometer (Thermo fisher Scientific, Waltham, USA) as already described.

### Western blotting

MDA-MB-231 cells were washed twice with ice-cold PBS and lysed with lysis buffer (137 mM NaCl, 2.7 mM KCl, 1 mM MgCl_2_, 1% TritonX-100, 10% v/v glycerol, 20 mM Tris-HCl, pH 7.8, 10 mM NaF, 1 mM EDTA, 0.5 mM Na_3_VO_4_, 0.2 mM PMSF and 1:100 Protease inhibitor cocktail (Sigma, P8340)). Total protein concentration was determined by BCA Protein Assay kit. Samples were adjusted for equivalent amount of proteins, dissolved in reducing Laemmli sample buffer (62.5 mM Tris-HCl, pH 6.8, 2% (w/v) sodium dodecyl sulfate (SDS), 10% (v/v) glycerol, 5% 2-mercaptoethanol, 0.002% bromophenol blue) and loaded on a 4–12% Bis-Tris polyacrylamide gel. Following electrophoresis, proteins were transferred to PVDF membrane using the Criterion system (Bio-Rad). Sample loading was assessed by membrane staining with Ponceau S (0.1% (w/v) Ponceau S in 5% (v/v) acetic acid). Then membranes were blocked in 5% (w/v) skimmed milk in TBS-T (20 mM Tris, 150 mM NaCl, 0.02% (v/v) Tween-20, pH 7.5) and incubated with primary antibodies overnight at 4 °C. Primary antibodies against p-ACC were diluted 1:5000 and primary antibodies against p-AMPK, total AMPK and GAPDG were diluted 1:10000. After washing, membranes were incubated with the appropriate secondary horseradish peroxidase-conjugated antibody, which was diluted between 1:100 and 1:25000 times. Immunoreactive proteins were detected with enhanced chemiluminescence using Agfa X-ray film. Densitometric analysis was performed with Quantity One 1-D Analysis Software (Bio-Rad).

### Statistical analysis

Results are presented as means of at least two independent experiments ± SEM. For statistical analysis, unpaired Student’s t-test or ANOVA, followed by an appropriate post hoc test, such as Bonferroni’s or Dunnett’s test, was performed using GraphPad Prism (v6; GraphPad Software, Inc., La Jolla, CA, USA). Synergism analysis was performed using two-way ANOVA in R software environment (version 3. 2. 2)^72^.

## Electronic supplementary material

Supplementary information
