# Supplementary material for: Combined treatment with Metformin and 2-deoxy glucose induces detachment of viable MDA-MB-231 breast cancer cells in vitro
Source: Sci Rep. 2017 May 11;7:1761. doi: 10.1038/s41598-017-01801-5 (PMC5431940; doi:10.1038/s41598-017-01801-5)
Supplement: Supplementary file 1 — Supplementary information [file 41598_2017_1801_MOESM1_ESM.pdf]

Supplementary information

Title: Combined treatment with Metformin and 2-deoxy glucose induces detachment of viable MDA-MB-231 breast cancer cells *in vitro*

Maruša Bizjak[1], Petra Malavašič[1], Klemen Dolinar[1,4], Jelka Pohar[2, 3], Sergej Pirkmajer[4], Mojca Pavlin[1,5]*

[1]Group for nano and biotechnological applications, Faculty of Electrical Engineering, University of Ljubljana, Ljubljana, Slovenia
[2]Department of Synthetic Biology and Immunology, National institute of Chemistry, Ljubljana, Slovenia
[3]Centre of Excellence EN-FIST, Ljubljana, Slovenia
[4]Institute of Pathophysiology, Faculty of Medicine, University of Ljubljana, Ljubljana, Slovenia
[5]Institute of Biophysics, Faculty of Medicine, University of Ljubljana, Ljubljana, Slovenia

```
## Analysis of Variance Table
##
## Response: attached_num
##              Df Sum Sq Mean Sq  F value    Pr(>F)
## Metf          1 2103.0  2103.0  29.6785 0.0006103 ***
## Two.DG        1 9314.3  9314.3 131.4471 3.032e-06 ***
## Metf:Two.DG   1  399.0   399.0   5.6313 0.0450326 *
## Residuals     8  566.9    70.9
## ---
## Signif. codes:  0 '***' 0.001 '**' 0.01 '*' 0.05 '.' 0.1 ' ' 1
```

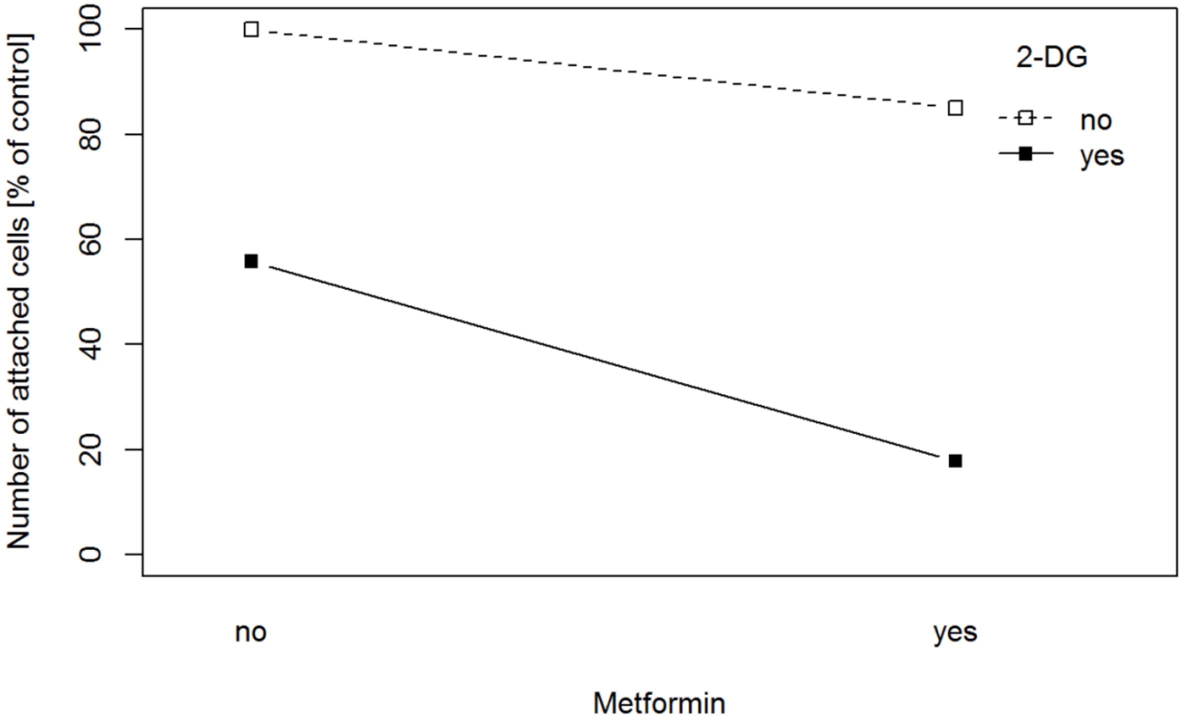

Supplementary Figure S1: Analysis of synergism between metformin and 2-DG on the percentage of attached cells

MDA-MB-231 cells were grown for three days in medium with (5.6 mM) glucose containing 5 mM metformin and/or 600 μM 2-DG. Number of attached cells was determined by Hoechst staining (Fig. 1B). Synergism analysis between metformin and 2-DG was performed using two-way ANOVA in R software environment (version 3. 2. 2). The effect of combined treatment with metformin and 2-DG on the number of attached cells is synergistic (combined effect is greater than additive effect of metformin and 2-DG) which is indicated with # in Fig. 1B.

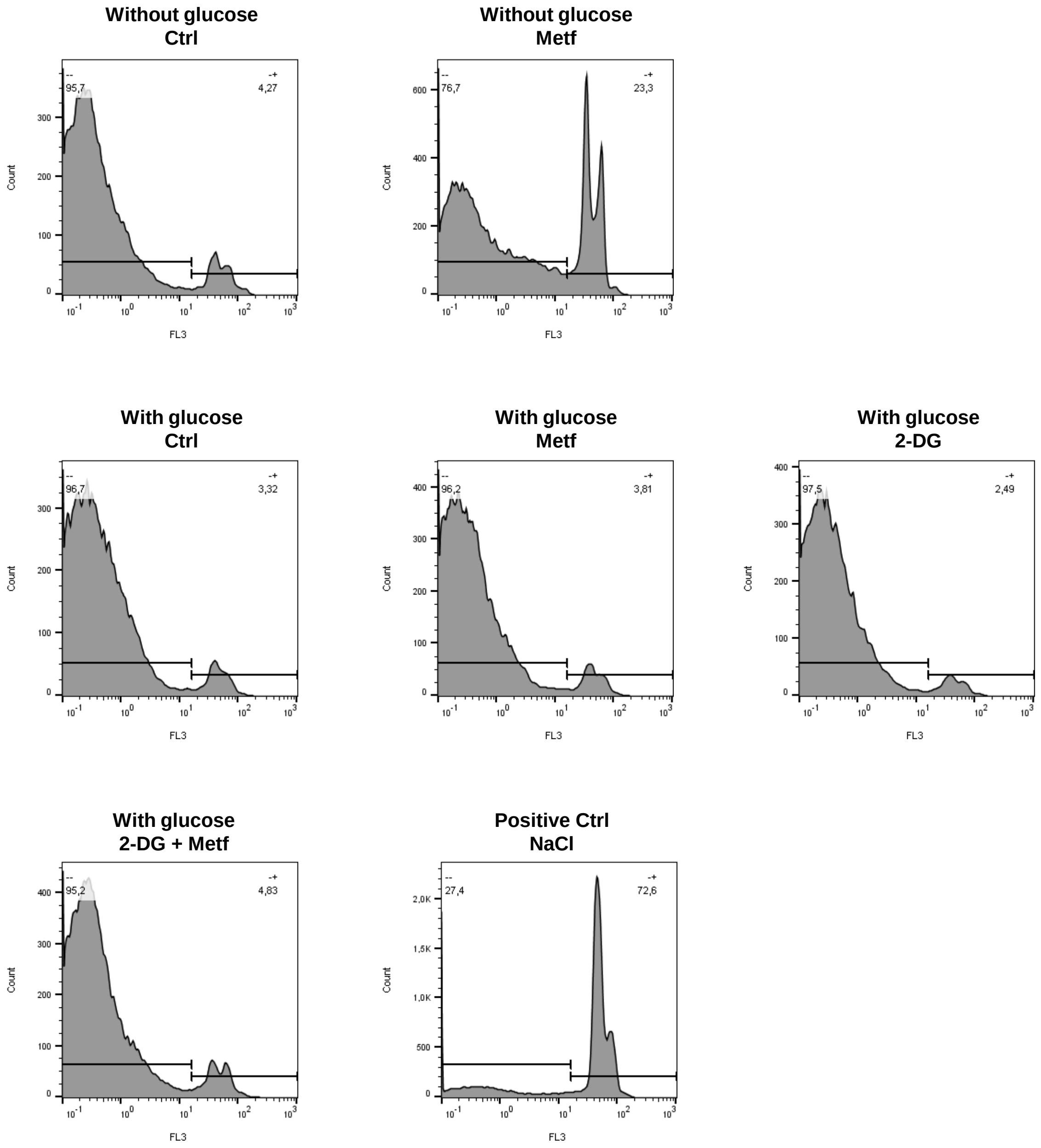

Supplementary Figure S2: Histograms showing distribution of two populations, dead and live MDA-MB-231 cells, using propidium iodide staining and flow cytometry

MDA-MB-231 cells were grown in medium with 5.6 mM glucose and treated with 5 mM metformin and 600 μM 2-DG for three days. As a positive control, MDA-MB-231 cells grown for two days in NaCl were used. Floating and adherent cells were joined and the percentage of dead cells was determined using propidium iodide staining and flow cytometer. Gates on histograms were set based on positive and negative control. Histograms of one independent experiment are shown. Mean and statistical analysis of three independent experiments is shown in Fig. 1 E, F.

```
## Analysis of Variance Table
##
## Response: floating.cells
##                  Df Sum Sq Mean Sq F value    Pr(>F)
## Metf_0.03         1  0.014   0.014  0.0170 0.8994349
## Two_DG            1 37.625  37.625 45.7709 0.0001428 ***
## Metf_0.03:Two_DG  1  0.167   0.167  0.2033 0.6640231
## Residuals         8  6.576   0.822
## ---
## Signif. codes:  0 '***' 0.001 '**' 0.01 '*' 0.05 '.' 0.1 ' ' 1
```

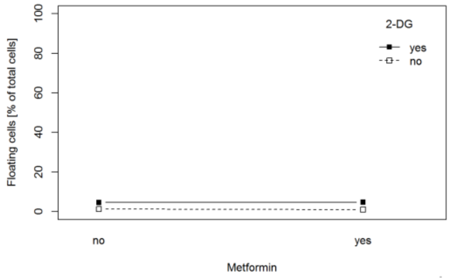


```
## Analysis of Variance Table
##
## Response: floating.cells
##                 Df  Sum Sq Mean Sq F value    Pr(>F)
## Metf_0.3         1  25.609  25.609  7.1186 0.0284469 *
## Two_DG           1 122.930 122.930 34.1708 0.0003847 ***
## Metf_0.3:Two_DG  1  28.754  28.754  7.9927 0.0222482 *
## Residuals        8  28.780   3.598
## ---
## Signif. codes:  0 '***' 0.001 '**' 0.01 '*' 0.05 '.' 0.1 ' ' 1
```

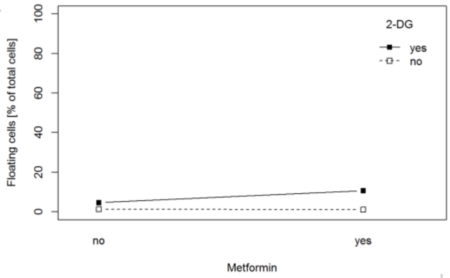


```
## Analysis of Variance Table
##
## Response: floating.cells
##               Df Sum Sq Mean Sq F value   Pr(>F)
## Metf_5         1 318.59  318.59  11.999 0.008519 **
## Two_DG         1 554.43  554.43  20.881 0.001827 **
## Metf_5:Two_DG  1 317.60  317.60  11.961 0.008587 **
## Residuals      8 212.42   26.55
## ---
## Signif. codes:  0 '***' 0.001 '**' 0.01 '*' 0.05 '.' 0.1 ' ' 1
```

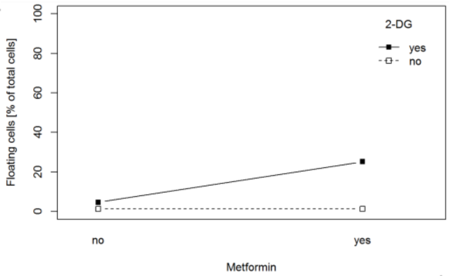


```
## Analysis of Variance Table
##
## Response: floating.cells
##                Df Sum Sq Mean Sq F value    Pr(>F)
## Metf_20         1 443.51  443.51  20.658 0.0018864 **
## Two_DG          1 578.73  578.73  26.957 0.0008305 ***
## Metf_20:Two_DG  1 336.05  336.05  15.653 0.0041983 **
## Residuals       8 171.75   21.47
## ---
## Signif. codes:  0 '***' 0.001 '**' 0.01 '*' 0.05 '.' 0.1 ' ' 1
```

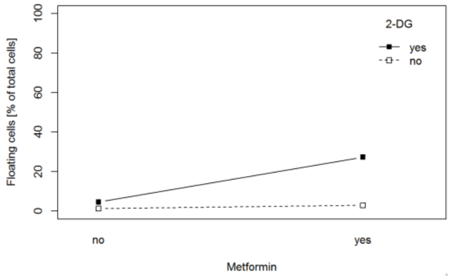

Supplementary Figure S3: Analysis of synergism between indicated concentrations of metformin and 600 µM 2-DG on the percentage floating cells

MDA-MB-231 cells were grown for two days in medium with (5.6 mM) glucose containing 600 µM 2-DG and/or indicated concentrations of metformin. The fraction of floating cells in each sample was determined using Countess cell counter. Synergism analysis between metformin and 2-DG was performed using two-way ANOVA in R software environment (version 3. 2. 2). The effect is synergistic between 0.3 mM, 5 mM and 20 mM metformin and 600 µM 2-DG (combined effect is greater than additive effect of metformin and 2-DG) which is indicated with # in Fig. 2B.

Day 1

```
## Analysis of Variance Table
##
## Response: floating_num
##              Df Sum Sq Mean Sq F value    Pr(>F)
## Metf          1  0.301   0.301  0.1711 0.6864201
## Two.DG        1 47.370  47.370 26.9704 0.0002244 ***
## Metf:Two.DG   1  0.497   0.497  0.2831 0.6043899
## Residuals    12 21.076   1.756
## ---
## Signif. codes:  0 '***' 0.001 '**' 0.01 '*' 0.05 '.' 0.1 ' ' 1
```

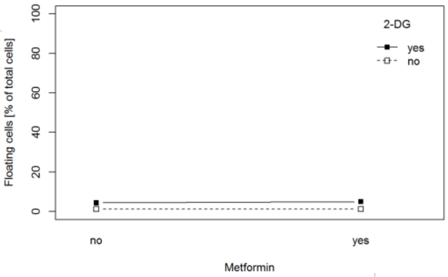

Day 2

```
## Analysis of Variance Table
##
## Response: floating_num
##              Df  Sum Sq Mean Sq F value    Pr(>F)
## Metf          1  539.86  539.86  14.404 0.0025517 **
## Two.DG        1 1039.64 1039.64  27.739 0.0001989 ***
## Metf:Two.DG   1  541.86  541.86  14.457 0.0025194 **
## Residuals    12  449.76   37.48
## ---
## Signif. codes:  0 '***' 0.001 '**' 0.01 '*' 0.05 '.' 0.1 ' ' 1
```

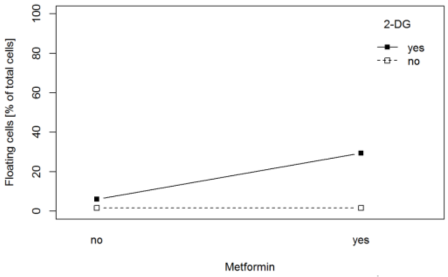

Day 3

```
## Analysis of Variance Table
##
## Response: floating_num
##              Df  Sum Sq Mean Sq F value    Pr(>F)
## Metf          1 2181.58 2181.58  99.029 3.775e-07 ***
## Two.DG        1 2965.46 2965.46 134.612 7.034e-08 ***
## Metf:Two.DG   1 2156.50 2156.50  97.890 4.018e-07 ***
## Residuals    12  264.36   22.03
## ---
## Signif. codes:  0 '***' 0.001 '**' 0.01 '*' 0.05 '.' 0.1 ' ' 1
```

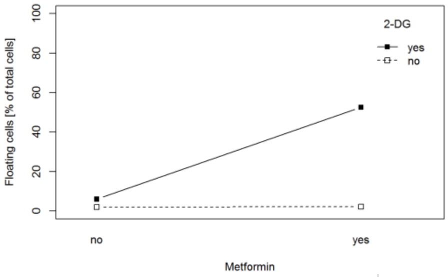

Supplementary Figure S4: Analysis of synergism between metformin and 2-DG on the percentage floating cells

MDA-MB-231 cells were grown for one to three days in medium with (5.6 mM) glucose containing 5 mM metformin and/or 600 µM 2-DG. The fraction of floating cells in each sample was determined using Countess cell counter. Synergism analysis between metformin and 2-DG was performed using two-way ANOVA in R software environment (version 3. 2. 2). The effect of combined treatment with metformin and 2-DG on the percentage of cells is synergistic on day 2 and 3 (combined effect is greater than additive effect of metformin and 2-DG) which is indicated with # in Fig. 2C.

```
## Analysis of Variance Table
##
## Response: Total_cells
##                 Df  Sum Sq  Mean Sq F value Pr(>F)
## Metf_0.03        1 0.09145 0.091452  1.0228 0.3415
## Two_DG           1 0.13255 0.132548  1.4824 0.2581
## Metf_0.03:Two_DG 1 0.13242 0.132418  1.4810 0.2583
## Residuals        8 0.71531 0.089414
```

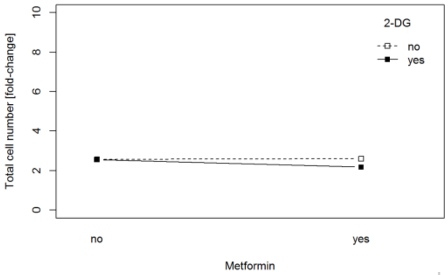


```
## Analysis of Variance Table
##
## Response: Total_cells
##                Df  Sum Sq Mean Sq F value  Pr(>F)
## Metf_0.3        1 0.44409 0.44409  6.4610 0.03461 *
## Two_DG          1 0.38132 0.38132  5.5478 0.04629 *
## Metf_0.3:Two_DG 1 0.38110 0.38110  5.5446 0.04634 *
## Residuals       8 0.54987 0.06873
## ---
## Signif. codes:  0 '***' 0.001 '**' 0.01 '*' 0.05 '.' 0.1 ' ' 1
```

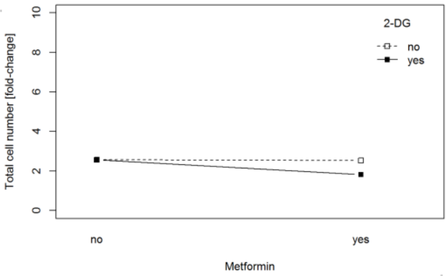


```
## Analysis of Variance Table
##
## Response: Total_cells
##              Df  Sum Sq Mean Sq F value   Pr(>F)
## Metf_5        1 2.18574 2.18574  20.218 0.002011 **
## Two_DG        1 1.75828 1.75828  16.264 0.003773 **
## Metf_5:Two_DG 1 1.75781 1.75781  16.260 0.003776 **
## Residuals     8 0.86486 0.10811
## ---
## Signif. codes:  0 '***' 0.001 '**' 0.01 '*' 0.05 '.' 0.1 ' ' 1
```

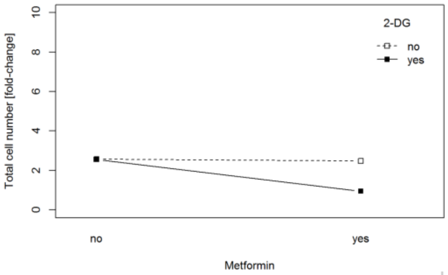


```
## Analysis of Variance Table
##
## Response: Total_cells
##               Df Sum Sq Mean Sq F value    Pr(>F)
## Metf_20        1 4.9959  4.9959  67.091 3.684e-05 ***
## Two_DG         1 0.8811  0.8811  11.832  0.008826 **
## Metf_20:Two_DG 1 0.8807  0.8807  11.828  0.008835 **
## Residuals      8 0.5957  0.0745
## ---
## Signif. codes:  0 '***' 0.001 '**' 0.01 '*' 0.05 '.' 0.1 ' ' 1
```

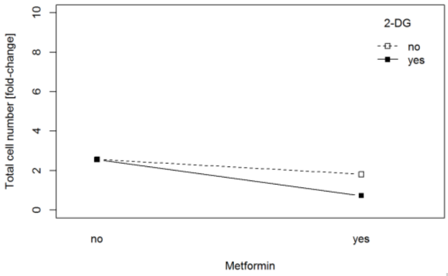

Supplementary Figure S5: Analysis of synergism between metformin and 2-DG on the number floating cells

MDA-MB-231 cells were grown for two days in medium with (5.6 mM) glucose containing 5 mM metformin and/or 600 μM 2-DG. Total number of cells in each sample was determined using Countess cell counter. Synergism analysis between metformin and 2-DG was performed using two-way ANOVA in R software environment (version 3. 2. 2). The effect is synergistic between 600 μM 2-DG and 0.3 mM, 5 mM and 20 mM metformin, which is indicated with # in Fig. 3B.

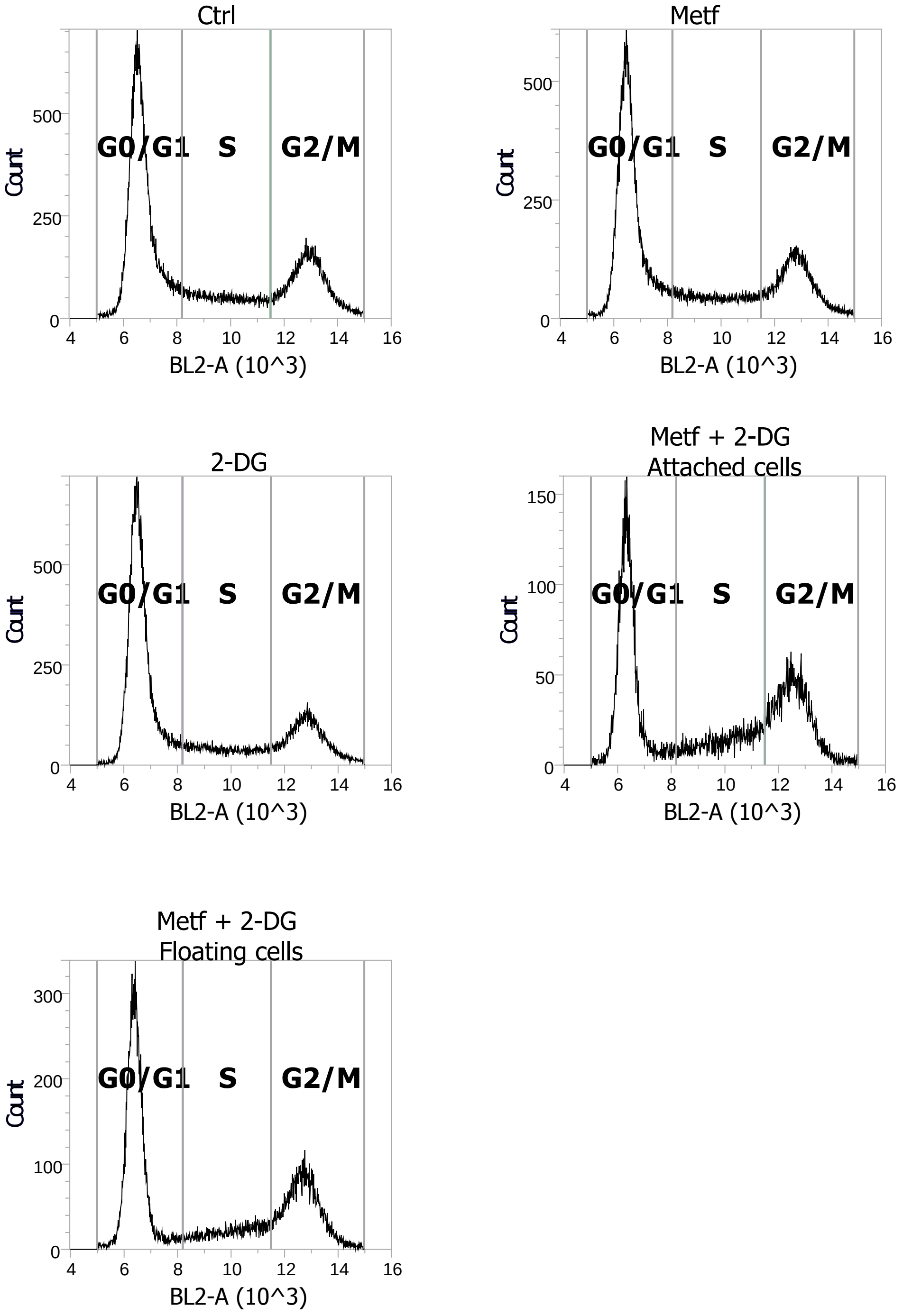

Supplementary Figure S6: Cell cycle analysis of MDA-MB-231 cells treated with metformin and 2-DG

MDA-MB-231 cells were cultured in medium with 5.6 mM glucose and treated with 5 mM metformin and/or 600 µM 2-DG for two days. Then cell cycle analysis was performed using propidium iodide staining and flow cytometry (see Materials and Methods). Histograms of one independent experiment are shown. Mean and statistical analysis of two independent experiments are shown in Fig. 3D.

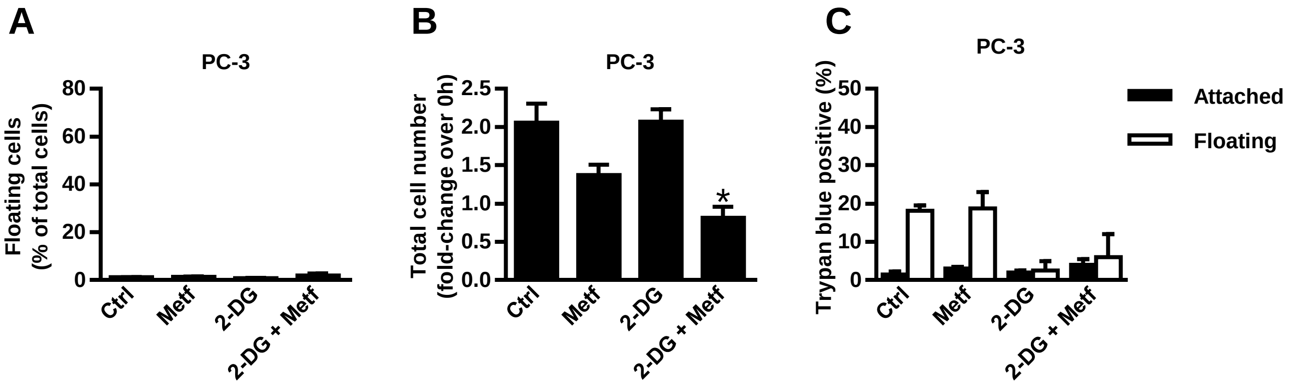

Supplementary Figure S7: PC-3 cells remain attached after combined treatment with metformin and 2-DG

(A, B, C) PC-3 cells were grown for two days in medium with 5.6 mM glucose and treated with 5 mM metformin and 600 μM 2-DG. The percentage of floating cells (A), total cell number (B) and the percentage of dead cells (C) were determined using trypan blue staining and Countess cell counter. Results are means±SEM (n=2). *$P=0.024$* vs. Ctrl.

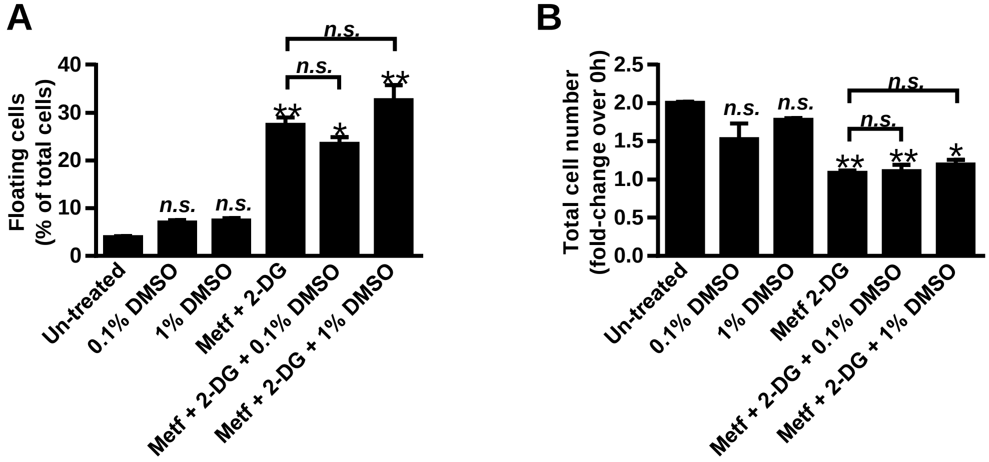

Supplementary Figure S8: DMSO alone does not prevent detachment of MDA-MB-231 cells treated with combination of metformin and 2-DG

(A, B) MDA-MB-231 cells were grown in medium with 5.6 mM glucose and treated with 5 mM metformin, 600 μM 2-DG, 0.1% DMSO (as used in experiments shown on Fig. 5A-E and Fig. 6) or 1% DMSO for two days. The percentage of floating cells (A) and total cell number (B) were deterimined using a Countess automated cell counter. Results are means±SEM (n=2). (A) *$P \leq 0.01$ vs. Ctrl; **$P \leq 0.0006$ vs. Ctrl. (B) *$P \leq 0.05$ vs Ctrl; **$P \leq 0.01$ vs Ctrl.

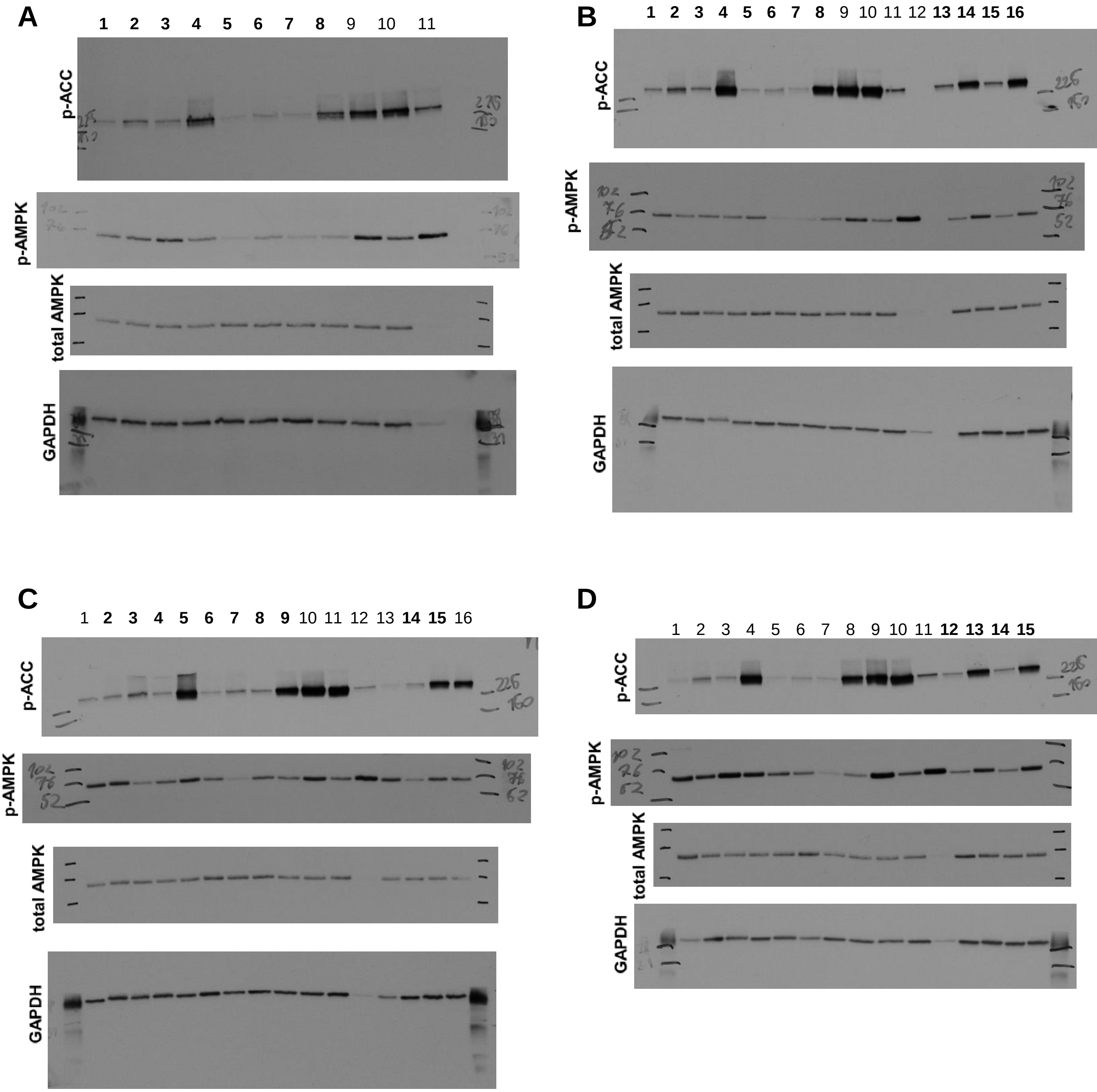

Supplementary Figure S9: AMPK activation is needed for detachment of viable MDA-MB-231 cells; original uncropped blots

(A-D) MDA-MB-231 cells were pre-treated with 5 μM Compound C (CC) in medium with glucose. Then 5 mM metformin and 600 μM 2-DG were added for additional 24 h. To determine the effects of anchorage-independent condition on AMPK activation, MDA-MB-231 cells were grown in a regular cell culture plate or in a cell culture plate covered with poly-HEMA (to prevent cell adhesion) for three days. Phosphorylation of AMPK and ACC and total AMPK level were determined by Western blot analysis. Sample names are as follows: A) from 1-11: Ctrl, Metf, 2-DG, 2-DG + Metf, CC, CC + Metf, CC + 2-DG, CC + Metf + 2-DG, 2 mM AICAR (2h), 2 mM AICAR + CC (2h), 2-DG + Metf floating cells; B) from 1-16: Ctrl, Metf, 2-DG, 2-DG + Metf, CC, CC + Metf, CC + 2-DG, CC + Metf + 2-DG, 2 mM AICAR (2h), 2 mM AICAR + CC (2h), 2-DG + Metf floating cells, empty, Ctrl (for floating cells), Floating cells, Ctrl (for floating cells, technical replicate), Floating cells (technical replicate); C) from 1-16: loading Ctrl, Ctrl, Metf, 2-DG, 2-DG + Metf, CC, CC + Metf, CC + 2-DG, CC + Metf + 2-DG, 2 mM AICAR (2h), 2 mM AICAR + CC (2h), 2-DG + Metf floating cells, loading Ctrl, Ctrl (for floating cells), Floating cells, Floating cells (technical replicate); D) from 1-15: Ctrl, Metf, 2-DG, 2-DG + Metf, CC, CC + Metf, CC + 2-DG, CC + Metf + 2-DG, 2 mM AICAR (2h), 2 mM AICAR + CC (2h), 2-DG + Metf floating cells, Ctrl (for floating cells), Floating cells, Ctrl (for floating cells, technical replicate), Floating cells (technical replicate). In final analysis (see Fig. 5), we did not include samples that were not evenly loaded or samples that did not have an evenly loaded control sample (as in D). Samples that were included in final analysis are indicated with bold numbers.

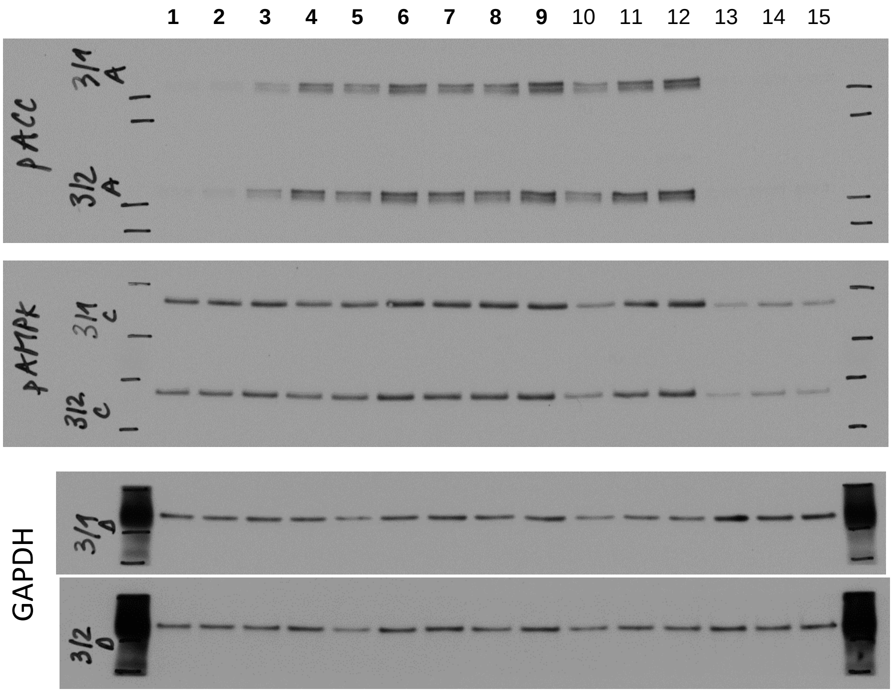

Supplementary Figure S10: AMPK activators AICAR and A-769662 do not induce detachment of viable MDA-MB-231 cells; original uncropped blots

MDA-MB-231 cells were cultured in the presence of 5 mM metformin, 600 µM 2-DG, 500 µM AICAR, 5 µM Compound C (CC) and 100 µM A-769662 and 5.6 mM glucose and in the absence of serum for 24 h. Phosphorylation of ACC and AMPK were determined using Western blot. Sample names are from 1-15 as follows: Ctrl, Metf, 2-DG, AICAR, AICAR + Metf, AICAR + 2-DG, A-769662, A-769662 + Metf, A-769662 + 2-DG, Metf + 2-DG, Metf + 2-DG + AICAR, Metf + 2-DG + A-769662, CC (24 h), CC + AICAR (24 h), CC + A-769662 (24 h). Blots from two independent experiments are shown. Samples that were included in final analysis (Fig. 6) are indicated with bold numbers.
